# DNA methylation entropy as a measure of stem cell replication and aging

**DOI:** 10.1186/s13059-023-02866-4

**Published:** 2023-02-16

**Authors:** Himani Vaidya, Hye Seon Jeong, Kelsey Keith, Shinji Maegawa, Gennaro Calendo, Jozef Madzo, Jaroslav Jelinek, Jean-Pierre J. Issa

**Affiliations:** 1 Coriell Institute for Medical Research, Camden, NJ 08013 USA; 2 Department of Neurology, Chungnam National University Hospital, Daejeon, South Korea; 3 Department of Pediatrics, University of Texas, MD Anderson Cancer Center, Houston, TX USA

**Keywords:** DNA methylation, Aging, Stem cell, Cell division, Epigenetic clock

## Abstract

**Background:**

Epigenetic marks are encoded by DNA methylation and accumulate errors as organisms age. This drift correlates with lifespan, but the biology of how this occurs is still unexplained. We analyze DNA methylation with age in mouse intestinal stem cells and compare them to nonstem cells.

**Results:**

Age-related changes in DNA methylation are identical in stem and nonstem cells, affect most prominently CpG islands and correlate weakly with gene expression. Age-related DNA methylation entropy, measured by the Jensen-Shannon Distribution, affects up to 25% of the detectable CpG sites and is a better measure of aging than individual CpG methylation. We analyze this entropy as a function of age in seven other tissues (heart, kidney, skeletal muscle, lung, liver, spleen, and blood) and it correlates strikingly with tissue-specific stem cell division rates. Thus, DNA methylation drift and increased entropy with age are primarily caused by and are sensors for, stem cell replication in adult tissues.

**Conclusions:**

These data have implications for the mechanisms of tissue-specific functional declines with aging and for the development of DNA-methylation-based biological clocks.

**Supplementary Information:**

The online version contains supplementary material available at 10.1186/s13059-023-02866-4.

## Introduction

Aging is the progressive decline in the physiology of an organism with time, and understanding the molecular and cellular hallmarks of aging [1] could lead to the prevention and treatment of age-related diseases. One of the least understood hallmarks of aging is epigenetic alterations. DNA methylation plays an important role in regulating gene expression, and its dysregulation during aging [2–5] and age-related disease [6] has been well-established. Studies of DNA methylation changes with age have shown that some CpG sites undergo hypomethylation with age [7, 8], especially at repetitive DNA sequences, which could lead to activation of retrotransposons, which, in turn, cause genomic instability with age; conversely, DNA hypermethylation with age occurs in gene promoter regions located within/near unmethylated CpG islands [9–12]. This phenomenon of either gaining or losing methylation at different genomic loci is known as methylation drift [11]. Age-related DNA methylation drift is highly conserved across different species, and this drift is inversely proportional to lifespan [12]. Studies have shown that twins living in the same environment acquire distinct age-related epigenetic changes, which indicates that it is a stochastic process rather than a genetic or environmental one [13–15].

Though the phenomenon of age-related epigenetic drift is well documented, there is little direct evidence for its underlying mechanisms. It was theorized that DNA methylation errors accumulate at specific CpG sites during replication in stem cells, which causes epigenetic drift that is then inherited by their daughter cells [16]. DNA methylation alterations have similar patterns in normal aging tissue and in cancer [17–21]. Because the addition of a methyl group on DNA occurs during DNA replication, the process of methylation drift with age is likely to be linked with stem cell division. Here, we test this hypothesis directly by examining stem cells vs. differentiated cells and examining tissues with different stem cell division rates. In addition, to develop a better understanding of epigenetic mosaicism, we use the concept of entropy and information theory to quantify methylation drift [22, 23].

## Results

### DNA methylation change with age is a stem cell phenomenon

**Fig. 1:**
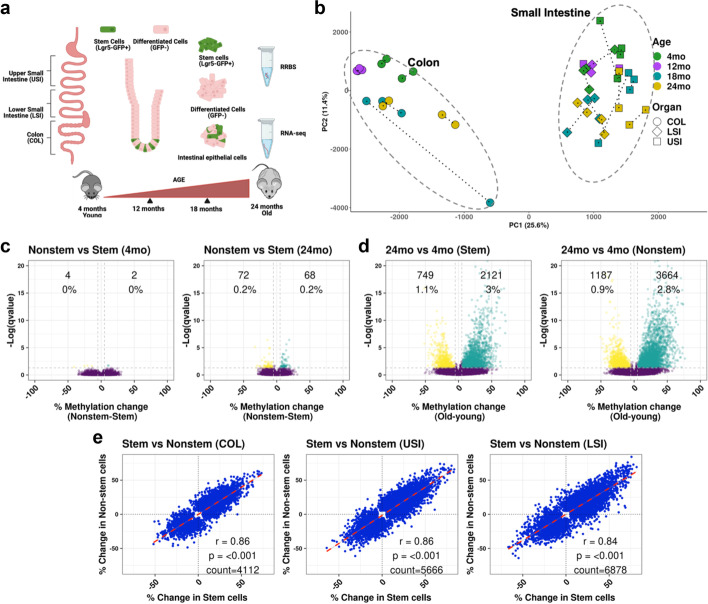
Methylation changes in stem cells correlate highly with their progeny. **a** Schematic diagram of experimental procedures. Mice were sacrificed at 4, 12, 18, or 24 months and intestines separated into the upper small intestine (USI), lower small intestine (LSI), and colon (COL). Stem cells were separated by sorting for Lgr5-GFP + cells. Reduced representation bisulfite sequencing (RRBS) and RNA-seq were done on the samples. **b** Principal component analysis (PCA) of 180,496 CpG sites common across 4 age groups and 3 tissues. Colon samples form a different cluster than small intestinal samples (PC1), and the spread along PC2 corresponds to an increasing age. **c** Volcano plots showing methylation differences between stem cells (Lgr5 − GFP +) and nonstem cells (Lgr5 − GFP −) in young and old mouse colon samples. Each volcano plot indicates the number and percent of CpG sites that are differentially methylated (Methylation change >  ± 5%, *q*-value < 0.05). **d** Volcano plots showing methylation differences between young (4 months) and old (24 months) samples in stem cells and nonstem cells. **e** Scatterplot of CpG sites that change significantly between 4 months versus 24 months in either stem cells, nonstem cells, or both in COL, USI, and LSI

To study stem cell-specific methylation patterns, we isolated Lgr5-GFP + intestinal stem cells and adjacent differentiated cells from Lgr5–EGFP–IRES–CreERT2 mice from mice aged 4, 12, 18, and 24 months. We selected the small intestine and colon (COL) as two separate tissue due to their functional difference and further divided the small intestine into two sections — upper small intestine (USI) and lower small intestine (LSI) — due to the difference in function of the duodenum and ileum and jejunum [24]. Isolated intestinal epithelial cells were FACS-sorted into stem cell (GFP positive) and differentiated cell (GFP negative) pools (4 months–4 mice/pool, 12 months–3 mice/pool, 18 and 24 months–2 mice/pool); these cells were then sequenced using reduced representation bisulfite sequencing (RRBS) to examine DNA methylation (Fig. 1a). Principal Component Analysis (PCA) of 180,496 CpG sites detected across all samples shows that the cells clustered with respect to tissue on PC1(25.6%), whereas variance with age can be seen on PC2 (Fig. 1b).

We used volcano plots to visualize differences in DNA methylation (if any). DNA methylation was identical (less than 0.1% CpG sites with methylation change) in the stem and nonstem cells in young (4 months) mice in the colon (Fig. 1c) and the small intestine (Additional file 1: Fig. S1.a-b). A similar analysis in old (24-month) animals also showed a high degree of similarity between stem and nonstem cells in the colon, though with greater discordance than in young animals in both the colon and the small intestine (Additional file 1: Fig. S1 c-d). The differences between the stem and nonstem cells in old animals were primarily in the non-CpG island, non-promoter genomic compartment (Additional file 2: Table S1). Next, we examined aging (24–4 months) separately in the stem and nonstem cell compartments. In colon stem cells, 3% of CpG sites gained DNA methylation in old mice, while 1.1% of CpG sites lost methylation (Fig. 1d, left). In the nonstem cell compartment, 2.8% of CpG sites gained DNA methylation, and 0.9% lost DNA methylation (Fig. 1d, right), with an overall change of 3–4% in both compartments with age. Comparable changes were seen in USI and LSI (Additional file 1: Fig. S1e-h). Across all comparisons, the genomic regions most likely to change with age were CpG islands, which became hypermethylated (Additional file 2: Table S2).

To determine whether the CpG sites that change with age are similar in stem and nonstem cells, we considered all CpG sites that changed in either stem cells, nonstem cells, or both. As shown in Fig. 1e, changes in methylation in stem and nonstem cells in the colon were highly correlated (*r* = 0.86, *p* < 0.001). We also saw similar results in USI (*r* = 0.86, *p* < 0.001) and LSI (*r* = 0.84, *p* < 0.001). We also compared DNA methylation change with age between different intestinal sections in stem and nonstem cells and found a significant correlation among them (Additional file 1: Fig. S1 i-n), which indicates that methylation change with age in different parts of the intestine occurs in the same CpG sites. Thus, DNA methylation changes with age reflect faithfully those alterations that happen in adult tissue-resident stem cells.

### Aging and differentiation target distinct genomic compartments

**Fig. 2:**
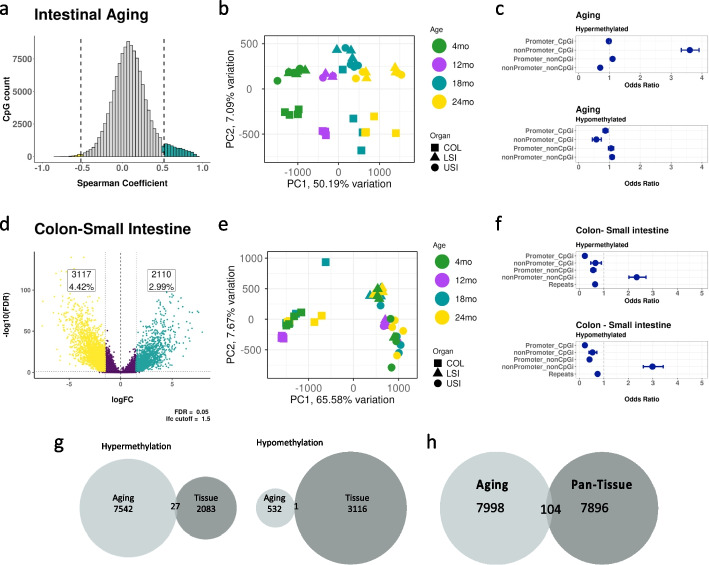
Aging and differentiation target distinct genomic compartments. **a** Histogram of Spearman correlation coefficients (*r*) derived from permutation analysis of 125,077 CpG sites( all samples, stem and Nonstem). Significantly (empirical *p*-value < 0.05, *r* >|0.5|) hypermethylated CpG sites are in red and hypomethylated sites are in green. **b** PCA plot constructed using the 8102 CpG sites from the permutation test that significantly change with age. **c** Odds ratios that CpG sites in given genomic regions (Promoter-CpGi, nonPromoter-CpGi, Promoter-nonCpGi, nonPromoter-nonCpGi) are more likely to gain methylation with age (top) or lose methylation with age (bottom). **d** Differential methylation analysis between the colon and small intestine (Upper small intestine + Lower small intestine) samples **e**. PCA plot constructed using the 5227 CpG sites that significantly change between the colon and small intestine in the differential methylation analysis. **f** Odds ratios that CpG sites in given genomic regions (Promoter-CpGi, nonPromoter-CpGi, Promoter-nonCpGi, nonPromoter-nonCpGi) are more likely to be hypermethylated in the colon (top) or be hypomethylated in the colon (bottom) compared to the small intestine. **g** Venn diagram showing the overlap of CpG sites that change significantly with age or significantly between colon vs small intestine, either sites that gain methylation (left) or sites that lose methylation (right). **h** Venn diagram showing the overlap of CpG sites that change with age significantly in the permutation analysis in the intestine with 8000 CpG sites common across multiple tissues (blood, heart, kidney, liver, lung, skeletal muscle, spleen, small intestine, and colon) with high standard deviation in methylation values in the same CpG sites

The data in Fig. 1 are based on a binary (old-young) analysis. We extended these findings across the lifespan, by incorporating data from mice at 12 and 18 months of age. To integrate the four time points in all three USI, LSI, and COL intestinal epithelial cells, and in both stem and nonstem cells, we used permutation testing on Spearman’s correlations (r) between methylation and age for the 125,077 CpG sites that had data across all ages with a minimum read coverage of 20 in at least 75% of the samples. Empirical *p*-values were based on 1000 random permutations. Based on the distributions of observed and permuted correlation coefficients (Additional file 1: Fig. S2a), we used a Spearman correlation cutoff of |0.5| and empirical *p*-value less than 0.05 to identify significantly changed sites. Overall, 7569 CpG sites (6.05%) were hypermethylated, and 533 CpG sites (0.43%) were hypomethylated (Fig. 2a), which confirms the results from the two-condition analysis.

PCA analysis of the 8102 sites that change significantly with age shows a predominant clustering based on age (PC1, 50.2% of the variance) and a minor clustering based on tissue of origin (PC2, 7.1% variance (Fig. 2b). This differs substantially from the PCA of all sites (Fig. 1b), where the predominant source of variance was tissue differentiation (PC1, 25.6%), followed by aging (PC2, 11.4%). To determine whether different genomic regions (Promoter-CpGi, nonPromoter-CpGi, Promoter-nonCpGi, nonPromoter-nonCpGi) were more likely to be affected by aging, we calculated odds ratios for each compartment in both the hyper- and hypo-methylated directions. As shown in Fig. 2c, nonPromoter-CpGi have 3.5 times the odds (95% CI 3.01–3.24) of gaining methylation with age, while nonPromoter-nonCpGi were less likely to gain methylation compared to the rest of the genome. PCA limited to significant nonPromoter-CpGi sites shows clustering solely on age with 58.3% of the variance in PC1 (Additional file 1: Fig. S2b). This specificity suggests that the CGI genomic compartment may yield better biomarkers of aging.

To determine whether the same sites are changing with respect to aging or tissue differentiation between the small intestine and colon, we compared sites that changed in methylation between them. We combined upper small intestine (USI) and lower small intestine (LSI) data as the small intestine. Differential methylation analysis showed that 2110 (3%) CpG sites significantly hypermethylated during differentiation between the colon and small intestine, and 3117 (4.4%) CpG sites are hypomethylated (Fig. 2d). PCA analysis of these significantly hyper- and hypo-methylated sites shows clustering based on tissue of origin with PC1 explaining 65.8% of the variance between the samples (Fig. 2e). Odds ratios calculated using significant CpG sites that change with tissue type in different genomic regions (Fig. 2f) showed that nonPromoter-nonCpGi sites had the greatest odds of gaining (2.3, 95% CI 2.02–2.7) or losing (3, 95% CI 2.6–3.4) methylation between the colon and the small intestine.

To determine whether the same CpG sites change with age and during tissue differentiation, we looked for common CpG sites that significantly change in the aging permutation analysis and sites that significantly change between colon vs. small intestine in the differential methylation analysis. We found very few sites in common (27 hypermethylated and 1 hypomethylated) between aging and tissue differentiation (Fig. 2g). Because the colon and small intestine are closely related tissues, we extended this analysis to other tissues, i.e., whole blood, heart, lung, liver, kidney, skeletal muscle and spleen (colon and small intestines included). We took common CpG sites across all tissues, allowing for 25% of the samples to have missing data. We calculated the standard deviation for each CpG site across these tissues and took the top 8000 CpG sites with the most deviation and tried to find overlapping CpG sites that are significant with age in our permutation analysis results. We found 104 CpG sites overlapping between the two, and the result is shown in the Venn diagram (Fig. 2h). Thus, these results demonstrate that tissue and age-related changes have distinct biological mechanisms. Studies show that sequencing depth of CpG sites affected CpG calling, and with higher sequencing depth (> 30), the estimation of their methylation levels had more concordance [25]. To test this, we ran the analysis with CpG sites with coverage > 30 and found similar results that were observed under coverage > 20. (Additional file 1: Fig. S3). However, using a coverage > 30 reduces the number of CpG sites analyzed from 125,077 to 70,547, which results in a 43% reduction in the number of sites analyzed.

### Gene expression changes with age and tissue differentiation in the intestine

**Fig. 3:**
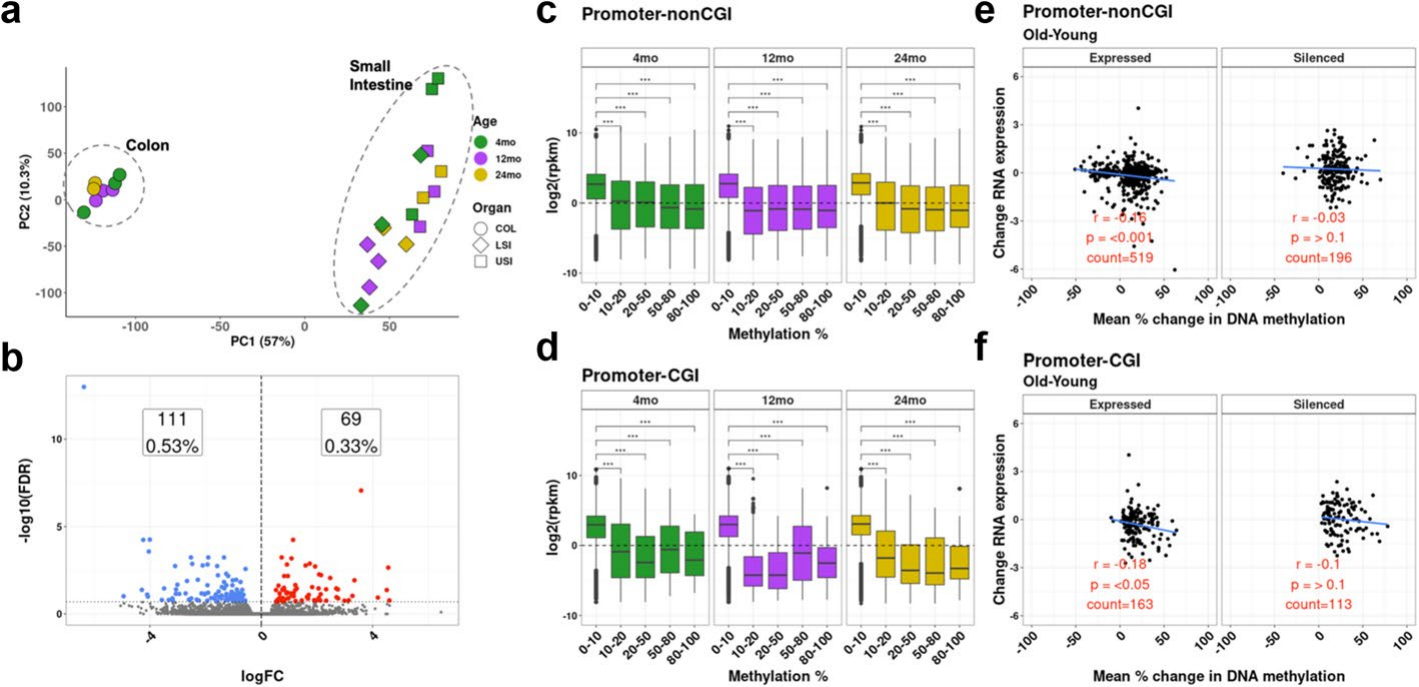
Gene expression changes with age and DNA methylation in the colon. **a** PCA plot constructed using all expressed genes in the intestinal epithelium of the upper small intestine (USI), lower small intestine (LSI), and colon (COL) at ages 4, 12, and 24 months. **b** Volcano plot showing differential gene expression between young (4 months) and old (24 months) colon samples. **c**, **d** Bar plots showing the relationship between DNA methylation (x-axis) and gene expression (average log2rpkm) on the *y*-axis in the nonCpG island (**c**) and promoter- CpG island (**d**) compartments. **e**, **f** Scatterplots showing the correlation between DNA methylation change and gene expression change for promoter CpG sites selected based on significance in the permutation analysis. Sites were further divided into those genes that are expressed in young mice (left) and those that are silenced at baseline (right). The plots show a weak but significant negative correlation between methylation change and expression change, for those genes that are expressed at baseline

DNA methylation analysis shows that aging in the intestine results in many CpG islands becoming hypermethylated. To see whether there are functional consequences to this dysregulation, we used RNA-seq to study intestinal epithelium from 4-month, 12-month, and 24-month-old mice, from the upper small intestine (USI), lower small intestine (LSI), and colon (COL). Principal component analysis shows that gene expression clusters based on tissue rather than age (Fig. 3a). Differential expression between the 4-month and 24-month colon (Fig. 3b) showed small changes in expression, with 21 genes that were significantly upregulated and 17 genes significantly downregulated (log2 Fold change > 1.5 and FDR < 0.05). Even fewer genes were differentially expressed between 4-month and 12-month colon samples (Additional file 1: Fig. S4 a-b), and there were similar results in the USI (Additional file 1 Fig. S4 c-e) and LSI (Additional file 1: Fig. S4 f–h) comparisons between the three age groups. By contrast, analysis of samples from different sections of the intestine showed many differentially regulated genes between USI, LSI, and COL (Additional file 1: Fig. S5 a-c).

To determine whether expression and methylation data were correlated, we plotted gene expression binned by methylation status (0–10%, 10–20%, 20–50%, 50–80%, and 80–100%) and analyzed separately promoters located outside or inside CpG islands. For promoters located in non-CpG island regions, expression of genes with low levels of methylation (0–10%) was higher than genes whose promoters had higher levels of methylation (Fig. 3c). This difference in gene expression with respect to DNA methylation was even more prominent in genes with promoters in CpG islands (Fig. 3d). Although the volcano plot analysis between 4-month and 24-month intestine did not show many differentially expressed genes, we explored changes in gene expression at the promoter-CpG sites that significantly changed methylation with age in the permutation analysis (Fig. 2). CpG sites that changed with age were further divided into those located in genes that are expressed or silenced in young mice. For expressed genes, we found a small but significant (Fig. 3e, f) inverse correlation between gene expression and DNA methylation change for CpG sites in promoter-nonCpG islands (*r* =  − 0.16, *p* < 0.01) and in promoter-CpG islands (*r* =  − 0.18, *p* < 0.05). As expected, those genes that are unexpressed at baseline showed no correlation between DNA methylation and gene expression change. These data show that age-related DNA methylation changes are much broader than gene expression changes, but the two processes do intersect for a few genes. To further explore RNA-seq data, we looked at non-canonical isoform changes between samples of different ages, but we did not find genes that express non-canonical isoforms with age (data not shown). However, due to the limited number of RNA-seq samples per age group, we do not have enough data to properly conduct this analysis.

### Aging but not differentiation leads to increasing entropy

**Fig. 4:**
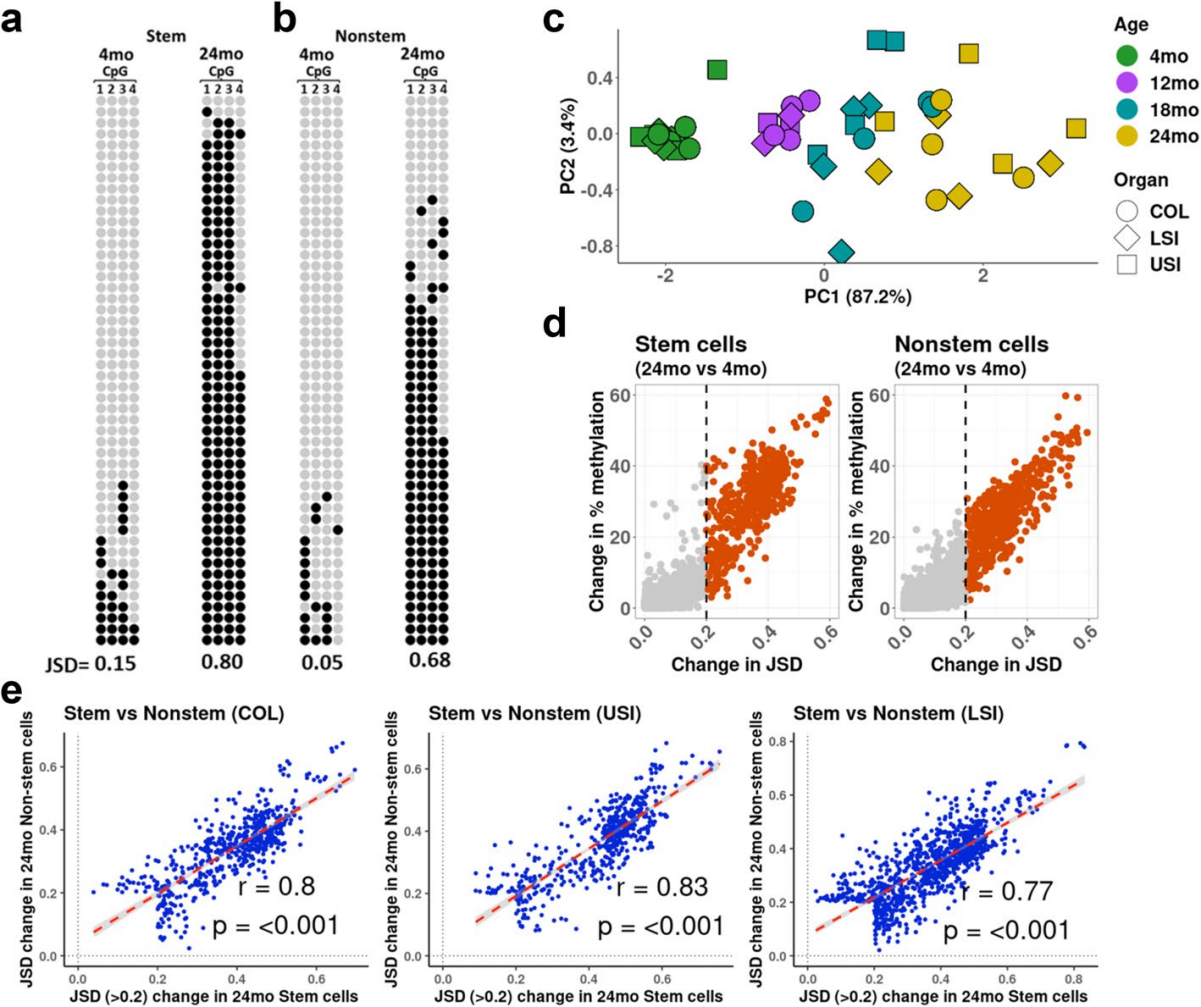
Aging but not differentiation leads to increasing entropy*.* a-b. Example methylation profiles of epialleles (4 CpGs on chr7 at positions 38,557,438, 38,557,457, 38,557,459, and 38,557,489) that have a large change in methylation entropy with age. Each row represents data from four consecutive CpG sites in a single sequenced allele (black: methylated, gray: unmethylated). **a** The left shows colon stem cells (Lgr5-GFP +) in a young (4 months) mouse vs the same loci at the right in old (24 months) mouse. (**b**) Same as **a**, but colon nonstem cells (Lgr5 − GFP −). **c** PCA plot constructed using Jensen-Shannon distances (JSDs) for 270 epialleles detected in all samples. The PCA shows clustering by age but not by tissue of origin. **d** Scatterplots showing the change in Jensen-Shannon distance (JSD) on the *x*-axis vs change in methylation on the *y*-axis in the colon between (left) young and old stem cells (Lgr5 − GFP +), and (right) young and old nonstem cells (Lgr5 − GFP −). **e** Scatterplot of CpG sites that have a JSD > 0.2 in old (24 months) in either stem cells, nonstem cells, or both in COL, USI, and LSI

Percent methylation provides an incomplete picture of DNA methylation changes because it does not consider allelic heterogeneity, also known as methylation entropy (an example of this is shown in Fig. 4a, b). We considered multiple methods to quantify entropy, including Shannon’s entropy [26] and combinatorial entropy [22]. However, those methods fail to consider the directionality of methylation change in the alleles because they treat all methylated alleles or all unmethylated alleles the same — both have an entropy of zero, which makes methylation entropy change harder to measure. To better quantify entropy, we used the change in epiallele distributions to calculate the Jensen-Shannon Distance (JSD) [22, 23], where samples are compared to a reference distribution (average JSD in 4-month samples). When the difference in the distance (JSD) between the reference and sample distribution equals or is closer to 0 there is no change in entropy, whereas a JSD of 1 refers to the greatest distance between reference and sample distribution where there is maximum change in entropy [23, 27].

In intestinal samples, 4-month-old colon samples have a narrow JSD distribution while, by 24 months, samples have a broad distribution, indicating a large dysregulation of the methylation program (Additional file 1: Fig. S6 a-f). There are 10,366 unique loci detected across all samples with a coverage greater than 40, but only 270 loci are present in all samples. We performed principal component analysis of JSD values in the 270 loci that present across all samples and found clustering solely on the basis of age (87.2%, PC1), with no clustering on the basis of tissue (Fig. 4c). This contrasts dramatically with Fig. 1b, which demonstrates that, while DNA methylation overall is primarily tissue-specific, DNA methylation entropy is exclusively an aging phenomenon.

To analyze the difference in JSD between stem cells and nonstem cells, we plotted change in entropy (*x*-axis) vs the change in methylation (*y*-axis). We found that there were very few loci changed in DNA methylation and entropy (JSD) between stem and nonstem cells (Additional file 1: Fig. S6 g-i).

in all three intestine compartments upper small intestine (USI), lower small intestine (LSI), and Colon (COL). In order to analyze the effect of aging on methylation entropy, we plotted change in entropy with age (*x*-axis) vs the change in methylation with age (*y*-axis) between stem and nonstem cell in old and young samples. We see large differences when comparing old and young samples in the colon, with 483 loci (23.8%) in stem cells and 589 loci (16.7%) in nonstem cells changing with age (Fig. 4d). Analysis for the USI (Additional file 1: Fig. S7 a-b) and LSI (Additional file 1: Fig. S7 c-d) sections showed similar results. To determine whether there is any change in methylation entropy between old (24-month) stem cell and nonstem cells, we considered all loci with JSD > 0.2 in either sample in the colon and Sl. As shown in Fig. 4e (left), changes in JSD in old stem and old nonstem cells were highly correlated (*r* = 0.8, *p* < 0.001). We saw similar results in USI (*r* = 0.83, *p* < 0.001) and LSI (*r* = 0.77, *p* < 0.001). Thus, there is no substantial difference in the disrupted entropy methylation patterns between stem cells and their daughter cells.

In our DNA methylation analysis, nonPromoter-CpGi had a higher odds of gaining methylation with age. To test this in the context of JSD, we divided the 270 loci present across all samples into CpG island and nonCpG island compartments. PCA analysis showed that both clustered with respect to age (Additional file 1: Fig. S8 a-b). We wanted to determine whether loci’s in CpG islands or nonCpG islands changed more with respect to age. We divided colon stem and nonstem cells in old and young samples into CpG island and non-CpG island compartments and analyzed change in JSD on the *x*-axis and change in DNA methylation on the *y*-axis. We find that CpG islands have more changes with an average of 45% of the loci’s changing with age in colon stem and nonstem cells, whereas only an average of 19% of loci in non-CpG islands change with age (Additional file 1: Fig. S8 c-h).

### Change in entropy in different tissues

There is controversy as to whether DNA methylation changes with age are strictly related to proliferation errors or are possibly secondary to other factors that also affect non-proliferating cells [20]. Given our data on DNA methylation entropy with age starting in stem cells, we reasoned that one test of its proliferation-dependence is to study tissues with different stem cell division rates. We therefore compared the change in entropy with age at 4 and 24 months between tissues that have very different stem cell division rates. Heart, kidney, and skeletal muscles have very low rates of stem cell division; other organs, such as the liver, spleen, and lung, have an increased rate of stem cell division, and the intestines have the highest rate. Similar to the intestine data, we looked at the distribution of JSD in 4-, 12-, and 24-month samples (Additional file 1: Fig. S9 a-g) for each tissue. In order to analyze the effect of aging on methylation entropy, we plotted change in entropy with age (*x*-axis) vs the change in methylation with age (*y*-axis) of each tissue. We found that in tissues with slow proliferative rates, such as the heart and kidney, there is very little change compared to intestinal data (Additional file 1: Fig. S9 h-n).

**Fig. 5:**
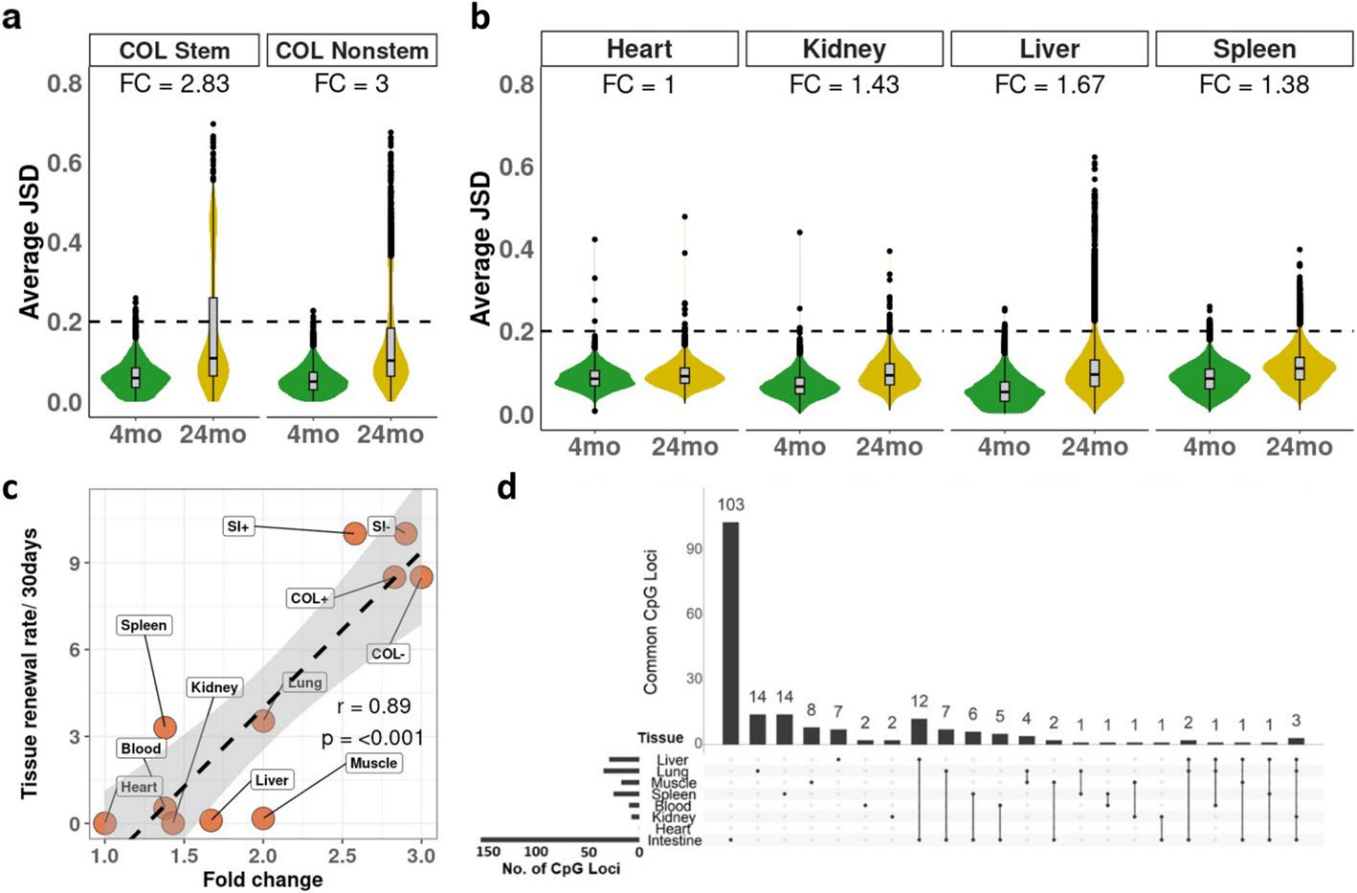
Change in entropy in different tissues. **a** Average Jensen-Shannon sistances for each locus (JSD) in young (4 months) and old (24 months) mice in the colon (COL) in both stem (Lgr5 − GFP +) and nonstem (Lgr5 − GFP −) cells (fold change calculation in the “Methods” section). **b** Same as **a** for the given tissue. **c** Scatterplot of fold change in JSD with age on the *x*-axis vs 30-day tissue renewal rate on the *y*-axis: COL +  = colon stem cells, COL −  = colon nonstem cells, SI +  = small intestine (USI + LSI) stem cells, SI −  = small intestine (USI + LSI) nonstem cells. **d** UpSet plots of commonly detected loci with Jensen-Shannon distance (JSD) > 0.2 in different organs (liver, lung, muscle, spleen, blood, kidney, heart, and intestines)

The distribution of entropy (JSD) with age (4-month vs 24-month) in the colon is shown in Fig. 5a, and data for the upper small intestine (USI) and lower small intestine (LSI) is shown in Additional file 1: Fig. S10 a-b, while Fig. 5b shows the distribution of entropy (JSD) in the heart, kidney, liver, and spleen. Our data show that between the old and young colon stem cells, a highly proliferative tissue, there is a threefold change in JSD distribution. In the Heart, a tissue with a very low proliferating rate, the fold change in the old versus the young is one, showing that minimal change occurs with age. We then plotted the fold change in entropy of different tissues against the 30-day tissue renewal rate (Fig. 5c). We took the overall cell division rates of these organs and calculated 30-day turnover rate (30-day turnover rate = 30 times the average turnover rate per day) for each organ (Additional file 2: Table S3). To calculate the whole organ cell turnover rate, we chose data sets where available with whole organ 3H-thymidine or Bromodeoxyuridine experiments. In cases where data was not available, we used stem cell division data for that organ (i.e., blood and muscle) because our hypothesis is that DNA methylation drift with age is a stem cell turnover phenomenon, and our data from colon and small intestine shows that this is true; thus, we assume that it is a similar case with other tissues as well. Results comparing fold change against 30-day cell turnover rates showed a very strong correlation (Pearson *r* = 0.89, *p*-value < 0.001), which suggests that methylation entropy is primarily a result of stem cell division. One difference between intestinal samples and other organs in that Intestinal data was derived from samples pooled during DNA extraction. In order to analyze whether pooling samples from different mice increases JSD, we took organs that had individual mouse samples and bioinformatically mimicked pooling. Results (not shown) obtained show that pooling decreases JSD and pooling more samples results in a decrease of JSD. Therefore, our data on pooled samples is possibly an underestimate of the true differences between individuals.

Finally, considering loci that were detected in all tissues, with a JSD > 0.2 in at least one tissue, we did not find loci that changed with age across all tissues and very few that changed in multiple tissues (Fig. 5d). In contrast, in the three sections of the intestine (which correspond to highly related tissues), most loci with JSD > 0.2 were overlapped (Additional file 1: Fig. S10 c). Thus, DNA methylation entropy change is highly tissue-specific, in addition to being very closely correlated with stem cell turnover. Lastly, in order to further justify our use of Jensen-Shannon Distance (JSD) to measure entropy, we also used Shannon’s entropy to measure change in entropy between 4-month and 24-month samples in different tissues, where Shannon’s entropy does not show significant changes between ages in the colon and other organs (Additional file 1: Fig. S10 d-e), whereas JSD shows considerable changes especially in the colon.

## Discussion

In this study, we found that most age-related DNA methylation drift is attributable to adult stem cell replication. Analysis of stem cells and differentiated cells in the same tissues show nearly identical age-related changes, and studies across nine different tissues show that stem cell turnover is the primary driver of methylation change with age. Furthermore, we find that the most specific methylation change linked to aging is an increase in entropy in the CpG island compartment.

Intestinal stem cells divide 3–4 times to differentiate into the different cell types that compose the intestinal epithelium [28]. We compared DNA methylation changes between stem cells and nonstem cells, and in young (4-month) mice, we found identical patterns. Old stem and nonstem cell samples, while still very similar, demonstrated more heterogeneity (Fig. 1d). This may be due to the possibility that older stem cells have a more unstable methylome. Alternately, some old stem cells with profoundly altered methylomes may have lost the ability to differentiate properly. The small differences in DNA methylation observed in old stem vs. nonstem cells could be explained if these profoundly altered stem cells are missing or under-represented in the differentiated cell compartment.

In this study, we found that using quantitative percent methylation to study aging gives an incomplete picture because it does not take epigenetic heterogeneity (allelic methylation) into account. Epigenetic heterogeneity can be defined as different patterns of DNA methylation in the same genetic region within different cells of a given tissue or origin [22, 29]. We used the principles of entropy, Jensen-Shannon distance, to study patterns of allelic methylation change with age. Jensen-Shannon distance measures the similarity between two probability distributions, with overlapping distributions (low entropy) having a low JSD and non-overlapping distributions (high entropy) having a high JSD [23]. Our results for the intestine showed that when compared between stem cells and nonstem cells of the same age, there was very little difference in entropy (Fig. 4e). When we compared the entropy change between 4-month and 24-month samples (in stem cell and nonstem cells), there were large changes in entropy (Fig. 4d, e). The importance of measuring entropy is clearest when comparing PCA plots using standard DNA methylation (Fig. 1b), the primary variation in data (PC1) is explained by tissue type. In marked contrast, PCA analysis of JSD values only identifies aging as a source of variability (Fig. 4c). Thus, using JSD instead of % methylation is preferable to study aging because it will not be influenced as much by changes in differentiation or cellular composition.

Using JSD, we find the clearest evidence to date that DNA methylation drift with age is a result of adult stem cell turnover. Tissues with low rates of cell division, i.e., heart and kidney, had much lower rates of entropy when compared to the intestine (Fig. 5a, b), while tissues with intermediate rates of cell division fell in between. This strongly argues that age-related DNA methylation changes are a result of random replication errors and are excellent biomarkers for stem cell age. Furthermore, the data are supported by the observation that chronic inflammation (which increases stem cell turnover) accelerates age-related methylation, while caloric restriction (which slows down metabolism and stem cell turnover) also slows down the accumulation of age-related methylation defects. We acknowledge that there is a limitation in our analysis because, in the colon and small intestine, the hierarchy is simple, whereas other tissues such as blood have a more complex hierarchy. In our analysis, we used three main types of tissues — non-proliferative (the best example being the heart), mildly proliferative (blood and lung for example), and very highly proliferative (colon and small intestinal epithelium). Aligning these is sufficient to give the strong correlation we observe (Fig. 5c), and our conclusion is that an increase in DNA methylation drift and entropy with age is a result of stem cell replication in adult tissues. However, not all tissues fit neatly in the scatterplot, but this likely reflects biology and unknowns. For example, the liver can be quiescent or proliferative depending on exposures over a lifetime. In some cases, the methylation data can be more accurate than the reported tissue renewal rates (blood and spleen likely have a shared ultimate stem cell, explaining similar methylation but different reported turnover rates). Also, methylation data for the organs was generated using whole organs and not purified cell types like epithelium. Some organs are constituted of cell types derived from different stem cells leading to skewing of results. It is possible that in tissues with complex hierarchy, there might be slightly different results, which is worth examining in the future.

Age-related methylation drift is evolutionarily conserved across species, and that methylation drift is inversely proportional to longevity [12]. Many groups have used these methylation changes to create epigenetic clocks that estimate biological age, with difference between biological age and estimated age correlating with disease and life expectancy [30–34]. Some of the clocks developed are used across many different tissues. Some studies have used methylation arrays to study changes in DNA methylation in mice; such arrays could provide a wider range of CpG sites to construct epigenetic clocks or to study tissue specificity [35]. It would be of interest to see if our differential methylation analysis results can be replicated using such arrays. However, one drawback of using arrays is that one cannot measure entropy using data generated from arrays. Our data suggest very little overlap in aging changes between distantly related tissues and between tissues that have very different stem cell proliferation rates. While clocks constructed by mixing groups of CpG sites specific for certain tissues may yield assays that work in different tissues, it may be preferable to use tissue-specific clocks for the most accurate results. Moreover, our data suggest that clocks that measure entropy may provide more accurate measurement of methylation age when compared to clocks based on % methylation.

## Conclusion

We have shown through PCA analysis that the overall DNA methylation pattern in intestinal epithelial cells is tissue specific. In the colon epithelium of young (4 months) mice, there are no DNA methylation differences between stem cells and nonstem cells, while this difference between stem and nonstem increases slightly (0.2%) in old (24 months) mouse colon, with small intestine showing similar results. However, with age (24 months vs 4 months), there is an overall DNA methylation change of 3–4% in both stem cells and nonstem cells and the changes in each cell type are highly correlated, leading us to the conclusion that DNA methylation drift with age in the intestinal epithelium is due to stem cell division. We extended these findings to include data from 12-month and 18-month mice using permutation analysis detecting 8102 CpG sites that significantly change with age and that the odds of DNA methylation change with age were the highest in non-promoter CpG islands. We performed a differential methylation analysis between the small intestine and colon irrespective of age and found that methylation changes in aging and in differentiation target distinct genomic compartments. RNA-seq analysis showed that DNA methylation change with age does affect overall RNA expression. In expressed genes, we found a small but significant inverse correlation between DNA methylation and gene expression, especially in CpG sites present in promoter-nonCpG islands.

We also quantified changes in entropy with age in the intestinal epithelium, using the Jensen-Shannon distance (JSD) and found that between stem cells and nonstem cells of the same age, there was very little difference in entropy, whereas there were large changes in entropy between 4 and 24 months in both stem cell and nonstem cells. PCA analysis using JSD showed age as the biggest source of variation between the sample, not cellular differentiation as was is shown in the DNA methylation PCA analysis. Since we first analyzed JSD from intestinal epithelium that has a high cellular turnover rate, we extended the comparison to other organs (blood, heart, kidney, liver, lung, skeletal muscle, and spleen) that have different turnover rates. We estimated the turnover rate for 30 days in each organ, calculated fold change in JSD with age, and saw that there was a high correlation between change in entropy and turnover rate offering further evidence for our conclusion that an increase in DNA methylation drift and entropy with age is a result of stem cell replication in adult tissues.

## Methods

### Mouse intestinal cells

Lgr5-EGFP-IRES-creERT2 (JAX Labs) mice were aged 4 months, 12 months, 18 months, and 24 months. At each time point, the small intestine and colon were harvested. The small intestine was bisected into two halves, upper and lower. Intestinal tissue sections were dissected longitudinally, washed with cold PBS, and cut into small pieces approximately 5 mm in length. The fragments were incubated for 20 min in 25 mL room temperature (15–25 °C) Gentle Cell Dissociation reagent (Stem Cell Technologies) on a rocking platform at 20 rpm. Tissue segments were gravity settled for 30 s and the supernatant was discarded. Tissue sections were resuspended in 10 ml cold PBS and vortexed for 5 s. After the tissue pieces were settled, the supernatant with the crypts was passed through a 70-μm filter. Vortexing and filtering were repeated 5 times. The collected crypts were centrifuged at 290 × g for 5 min. Supernatant was discarded and the pellet was resuspended in 2 ml Tryple Express with 200 μl DNAseI (NEB) and incubated at 37 °C for 20 min with agitation every 5 min. The dissociated cells were passed through a 20 μm filter and then sorted (BD FACS Aria II & Sony SH800) into GFP + and GFP − pools to separate stem cells and nonstem cells, respectively.

### Non-intestinal mouse tissues

Whole mouse organs or tissues from Lgr5-EGFP-IRES-creERT2 mice were collected along with the intestinal sections. They were minced using a scalpel and incubated at 50 °C in 2% SDS and 25 mM EDTA for 3 days.

### DNA extraction

Collected cell pools for all tissues were lysed in 2% SDS and 25 mM EDTA overnight. Ammonium acetate (10 M) was added to make the precipitate of SDS with proteins and was removed by centrifugation at 12,000 rpm. Next, DNA was precipitated using isopropanol, washed with 70% ethanol, and dissolved in dissolved in LTET (Tris 10 mM, EDTA, 0.1 mM + Tween 20,0.1%). DNA concentration was measured using Qubit dsDNA Broad Range kit (Thermo Fisher, # Q32850).

### Reduced representation bisulfite sequencing (RRBS)

Two micrograms of DNA were digested with 100 U of *Msp*I endonuclease (NEB, #R0106) at 37 °C for 3.5 h. Klenow fragment (3′ > 5′ exo-) DNA polymerase (NEB, #M0212) was used to fill in 3′ recesses and create 3′ dA overhangs. Size selection by AMPure XP beads (Beckman, # A63881) with 2X bead solution to DNA volume was performed. Methylated sequencing adapters (NEB, #E7535) were ligated to the DNA fragments using T4 DNA ligase (NEB #M0202) at 16 °C overnight. Adapters were cleaved with by incubation with USER enzyme (NEB #M5505) at 37 °C for 15 min. Dual size selection was done by AMPure XP beads to remove DNA fragments greater than 500 bp and less than 150 bp. Purified DNA fragments were bisulfite converted using the EpiTect Bisulfite Conversion Kit (Qiagen, #59,104). The bisulfite-converted fragments were cleaned up using 3 × AMPure beads to DNA ratio. We used NEBNext Index i7 primers (NEB #7335, #7500) to amplify the libraries with EpiMark Hot Start Taq (NEB, #M0490) for 15 cycles. Resulting library concentrations were measured using the Qubit dsDNA High Sensitivity kit (Thermo Fisher, # Q32851). Agilent Bioanalyzer was used to determine the size distribution and molarity of the libraries using the Agilent High Sensitivity DNA kit (Agilent, #5067–4626). The libraries were pooled to a concentration of 10 nM and spiked 25% with phiX (Illumina, #FC1103001) to ensure sequence diversity. Sequencing was performed over several years at multiple facilities.

### RNA extraction and sequencing

With the exception of sorting, pooled intestinal cells were extracted as above. Pellets were lysed in 1 mL TRIzol (Thermo Fisher, # 15,596,026) and 100 µL of bromo-chlorophenol (Fisher, # AC173530050) was added. Phase separation was done by centrifugation at 12,000 g for 15 min at 4 °C. Supernatant containing RNA was collected, RNA precipitated with an equal volume of isopropanol, and washed with 1 ml 75% ethanol. The RNA pellet was collected by centrifuging at 7500 g for 5 min and dissolving in 30 μl nuclease-free water. RNA concentration was determined with Qubit RNA Broad range kit (Thermo Fisher, # Q10210) and RNA integrity score (RIN) was determined using Agilent RNA 6000 nano kit (Agilent, # 56,067–1511). RNA was sent to BGI, who performed library preparation and sequencing.

### Data processing

Sequenced RRBS libraries were trimmed using TrimGalore and the Bismark Bisulfite Read Mapper and Methylation Caller were used to align reads to the mm9 genome and call CpG methylation. Data were filtered for a minimum coverage of 20 reads at each CpG. RNA libraries were trimmed using trimmomatic, then aligned to the mm9 genomes and genes counted using STAR. Differential expression analysis was performed in R using the edgeR with standard parameters.

### Differential methylation

For two condition comparisons, differential methylation was calculated using the methylKit R package. Data were corrected for overdispersion, then differential methylation was called with methylKit standard parameters, using chi-square with *p*-values corrected for multiple testing using the SLIM method [36]. Statistical significance was calculated by a chi-square test and corrected for overdispersion. CpG sites were filtered for an absolute methylation difference of greater than 5% and a corrected *p*-value of less than 0.05 to select CpG sites showing methylation changes. All sample differential methylation was tested using a permutation test. Data were stripped of their identifiers, randomly shuffled 1000 times, and then the Spearman correlation between these randomly shuffled values and percent methylation was calculated. Empirical *p*-values were calculated using the formula $p= \frac{r}{n}$ where p is the empirical *p*-value, *r* is the number of replicates with a Spearman correlation greater than or equal to the given correlation, and *n* is the total number of permutations performed. CpG sites were filtered for |*r*|≥ 0.5 and empirical *p*-value < 0.05 to select CpG sites showing methylation changes.

### Methylation entropy

Methclone [22] was used to find epialleles, defined as four CpGs within 60 bp of each other with a minimum coverage of 40 reads. The Jensen-Shannon distance was calculated using the epiallele frequencies, with 0 CpGs, 1 CpG,…, 4 CpGs methylated, to quantify the disorder present in the epialleles, using the formula

$$\begin{array}{c}d=\sqrt{\frac{D\left(P_L^{(1)},\;Q\right)+D\left(P_L^{(2)},\;Q\right)}2}\\\mathrm D\left(P,\;Q\right)=\sum\limits_\ell P(\ell)log_2\left(\frac{P\mathit(\ell\mathit)}{Q\mathit(\ell\mathit)}\right)\end{array}$$

where $\mathcal{l}$ is the frequency of methylated CpGs on the epiallele; $Q$ is the reference distribution, for example, average allele frequency in young mouse colon cells; and *P* is the sample distribution, for example, 24-month-old mouse colon stem cells. If the reference and sample distributions do not overlap JSD equals 1, if they overlap completely then JSD equals 0, and with intermediate levels of overlap the value ranges between 0 and 1.

### Differential isoform

Raw paired-end RNA-seq reads were quality trimmed using trim_galore with default settings. Following trimming, reads were quantified using Salmon v1.9 to the GRCm39 transcriptome and setting the number of bootstrap replicates to 30 in order to estimate the technical variance in abundance estimates prior to differential isoform usage analysis. The R package fishpond was then used to scale counts from bootstrap replicates and estimate isoform proportions. Differential isoform usage was then performed using the swish method in order to account for uncertainty in transcript abundances and identify transcripts where the isoform proportions change across condition.

### Statistics

Odds ratios (OR) were calculated for changes in methylation at specific genomic locations (CpG-Promoter, CpG-NonPromoter, nonCpG-Promoter, nonCpG-nonPromoter) using a 2 × 2 contingency table comparing CpGs in the given location to all other CpGs and Fisher’s exact test.

Differences between the JSD of old and young samples were assessed using Levene’s test of equal variance and corrected for multiple testing using Bonferroni correction.

JSD fold change was calculated by taking the means of the different groups divided by the mean in the reference group using the formula:

$$\mathrm{fold change}=\left(\frac{\frac{\Sigma {JSD}_{\mathrm{old}}}{n}-\frac{\Sigma {JSD}_{\mathrm{young}}}{n}}{\frac{\Sigma {JSD}_{\mathrm{young}}}{n}}\right)$$

where JSD is the Jensen-Shannon distance for the given group and n is the number of epialleles.

All plots were generated in *R* using the ggplot2 package.

## Supplementary Information

**Additional file 1: **Contains all supplementary figures and supplementary methods.

**Additional file 2:** Contains all supplementary tables.

**Additional file 3:** Review history.

## Data Availability

Sequence data from this article can be found in the NCBI GEO under accession number GSE213723 (37). Further information and requests for resources and reagents should be directed to and will be fulfilled by the Lead Contact, Jean-Pierre J. Issa, MD (jpissa@coriell.org).
