# Author Correction: DNA methylation entropy as a measure of stem cell replication and aging

**DOI:** 10.1186/s13059-023-02943-8

**Published:** 2023-04-30

**Authors:** Himani Vaidya, Hye Seon Jeong, Kelsey Keith, Shinji Maegawa, Gennaro Calendo, Jozef Madzo, Jaroslav Jelinek, Jean-Pierre J. Issa

**Affiliations:** 1 Coriell Institute for Medical Research, Camden, NJ 08013 USA; 2 Department of Neurology, Chungnam National University Hospital, Daejeon, South Korea; 3 Department of Pediatrics, University of Texas, MD Anderson Cancer Center, Houston, TX USA

**Correction: Genome Biol 24, 27 (2023)**

**https://doi.org/10.1186/s13059-023-02866-4**

Following the publication of the original paper [1], the authors reported an error in Fig. 2.

**Fig. 2:**
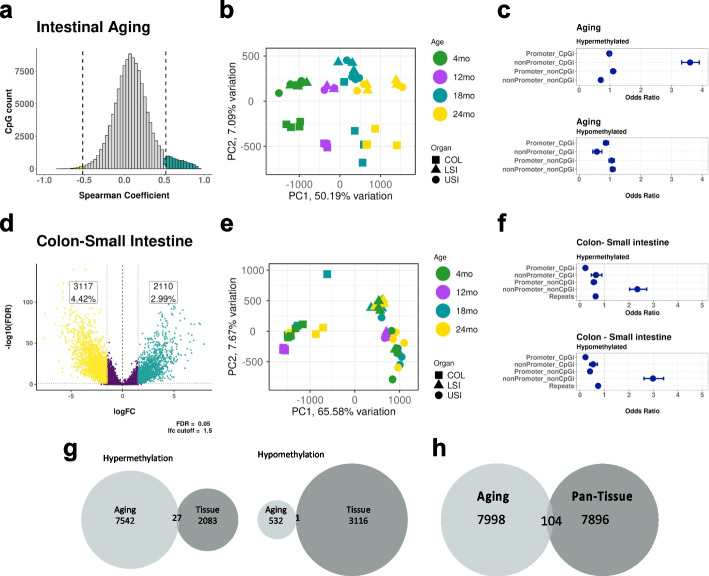
Aging and differentiation target distinct genomic compartments. **a** Histogram of Spearman correlation coefficients (*r*) derived from permutation analysis of 125,077 CpG sites( all samples, stem and Nonstem). Significantly (empirical *p*-value < 0.05, *r* >|0.5|) hypermethylated CpG sites are in red and hypomethylated sites are in green. **b** PCA plot constructed using the 8102 CpG sites from the permutation test that significantly change with age. **c** Odds ratios that CpG sites in given genomic regions (Promoter-CpGi, nonPromoter-CpGi, Promoter-nonCpGi, nonPromoter-nonCpGi) are more likely to gain methylation with age (top) or lose methylation with age (bottom). **d** Differential methylation analysis between the colon and small intestine (Upper small intestine + Lower small intestine) samples **e**. PCA plot constructed using the 5227 CpG sites that significantly change between the colon and small intestine in the differential methylation analysis. **f** Odds ratios that CpG sites in given genomic regions (Promoter-CpGi, nonPromoter-CpGi, Promoter-nonCpGi, nonPromoter-nonCpGi) are more likely to be hypermethylated in the colon (top) or be hypomethylated in the colon (bottom) compared to the small intestine. **g** Venn diagram showing the overlap of CpG sites that change significantly with age or significantly between colon vs small intestine, either sites that gain methylation (left) or sites that lose methylation (right). **h** Venn diagram showing the overlap of CpG sites that change with age significantly in the permutation analysis in the intestine with 8000 CpG sites common across multiple tissues (blood, heart, kidney, liver, lung, skeletal muscle, spleen, small intestine, and colon) with high standard deviation in methylation values in the same CpG sites

The original article [1] has been corrected.
