# Supplementary material for: DNA methylation entropy as a measure of stem cell replication and aging
Source: Genome Biol. 2023 Feb 16;24:27. doi: 10.1186/s13059-023-02866-4 (PMC9933260; doi:10.1186/s13059-023-02866-4)
Supplement: Supplementary file 1 — Additional file 1: Contains all supplementary figures and supplementary methods. [file 13059_2023_2866_MOESM1_ESM.pdf]

Fig. S1

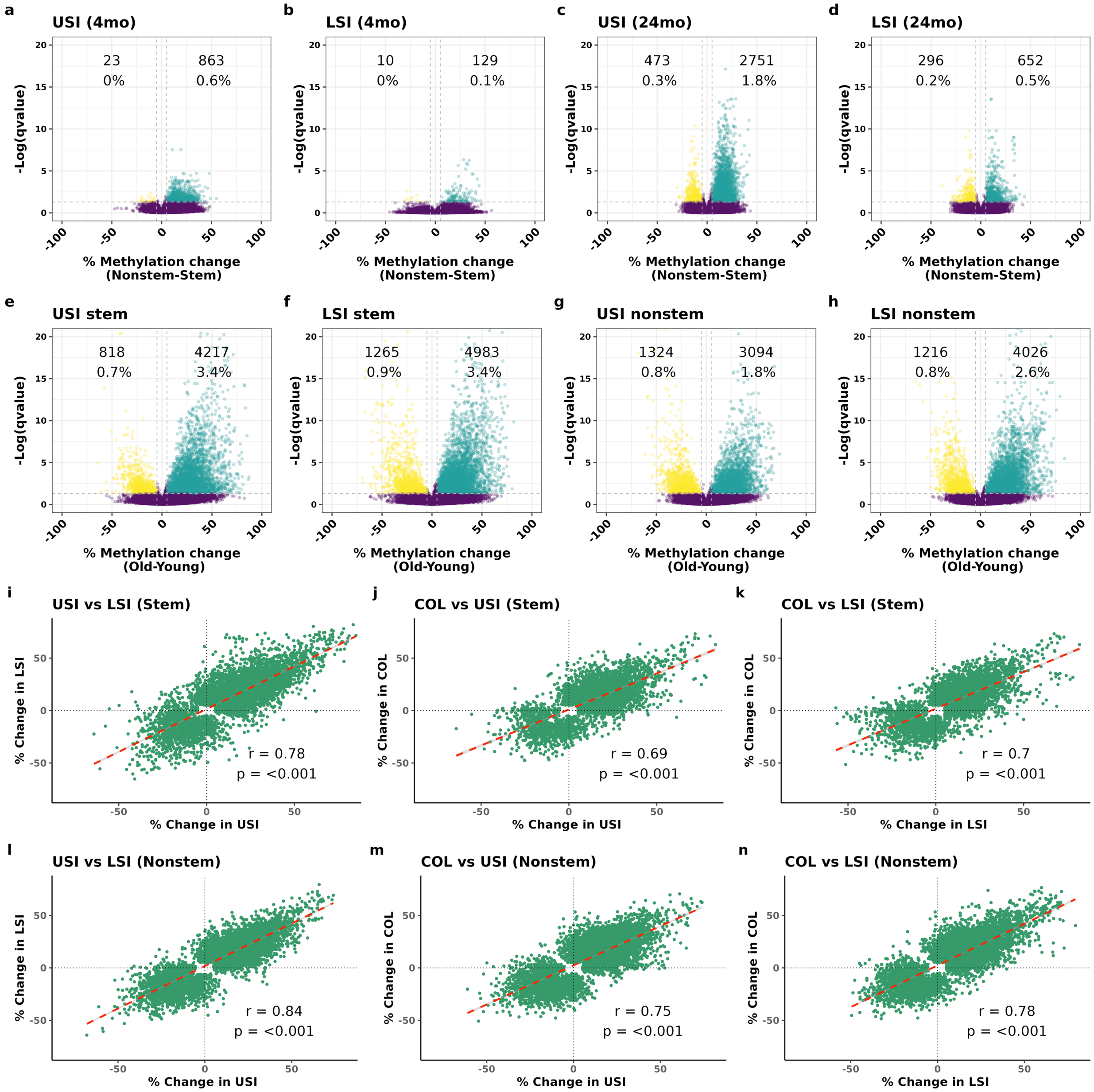

a
USI (4mo)
23
0%
863
0.6%
b
LSI (4mo)
10
0%
129
0.1%
c
USI (24mo)
473
0.3%
2751
1.8%
d
LSI (24mo)
296
0.2%
652
0.5%
-Log(qvalue)
% Methylation change
(Nonstem-Stem)
e
USI stem
818
0.7%
4217
3.4%
f
LSI stem
1265
0.9%
4983
3.4%
g
USI nonstem
1324
0.8%
3094
1.8%
h
LSI nonstem
1216
0.8%
4026
2.6%
% Methylation change
(Old-Young)
i
USI vs LSI (Stem)
r = 0.78
p = <0.001
% Change in LSI
% Change in USI
j
COL vs USI (Stem)
r = 0.69
p = <0.001
% Change in COL
% Change in USI
k
COL vs LSI (Stem)
r = 0.7
p = <0.001
% Change in COL
% Change in LSI
l
USI vs LSI (Nonstem)
r = 0.84
p = <0.001
% Change in LSI
% Change in USI
m
COL vs USI (Nonstem)
r = 0.75
p = <0.001
% Change in COL
% Change in USI
n
COL vs LSI (Nonstem)
r = 0.78
p = <0.001
% Change in COL
% Change in LSI

Fig. S2

a

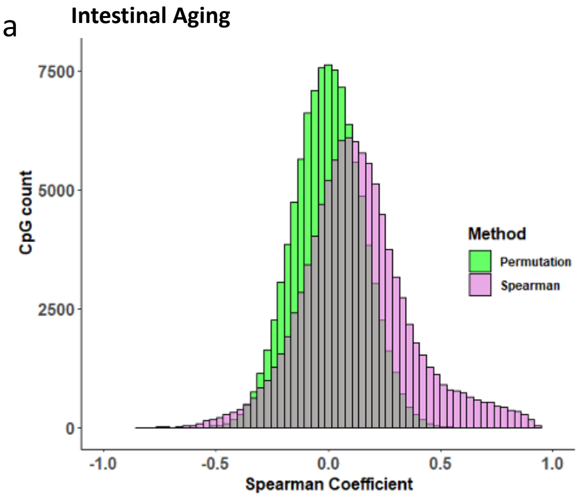
Intestinal Aging
CpG count
7500
5000
2500
0
-1.0
-0.5
0.0
0.5
1.0
Spearman Coefficient
Method
Permutation
Spearman

b

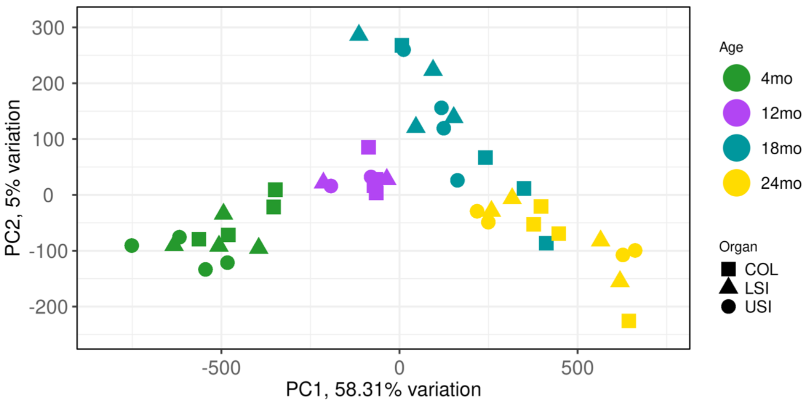
300
200
100
0
-100
-200
PC2, 5% variation
-500
0
500
PC1, 58.31% variation
Age
4mo
12mo
18mo
24mo
Organ
COL
LSI
USI

Fig. S3

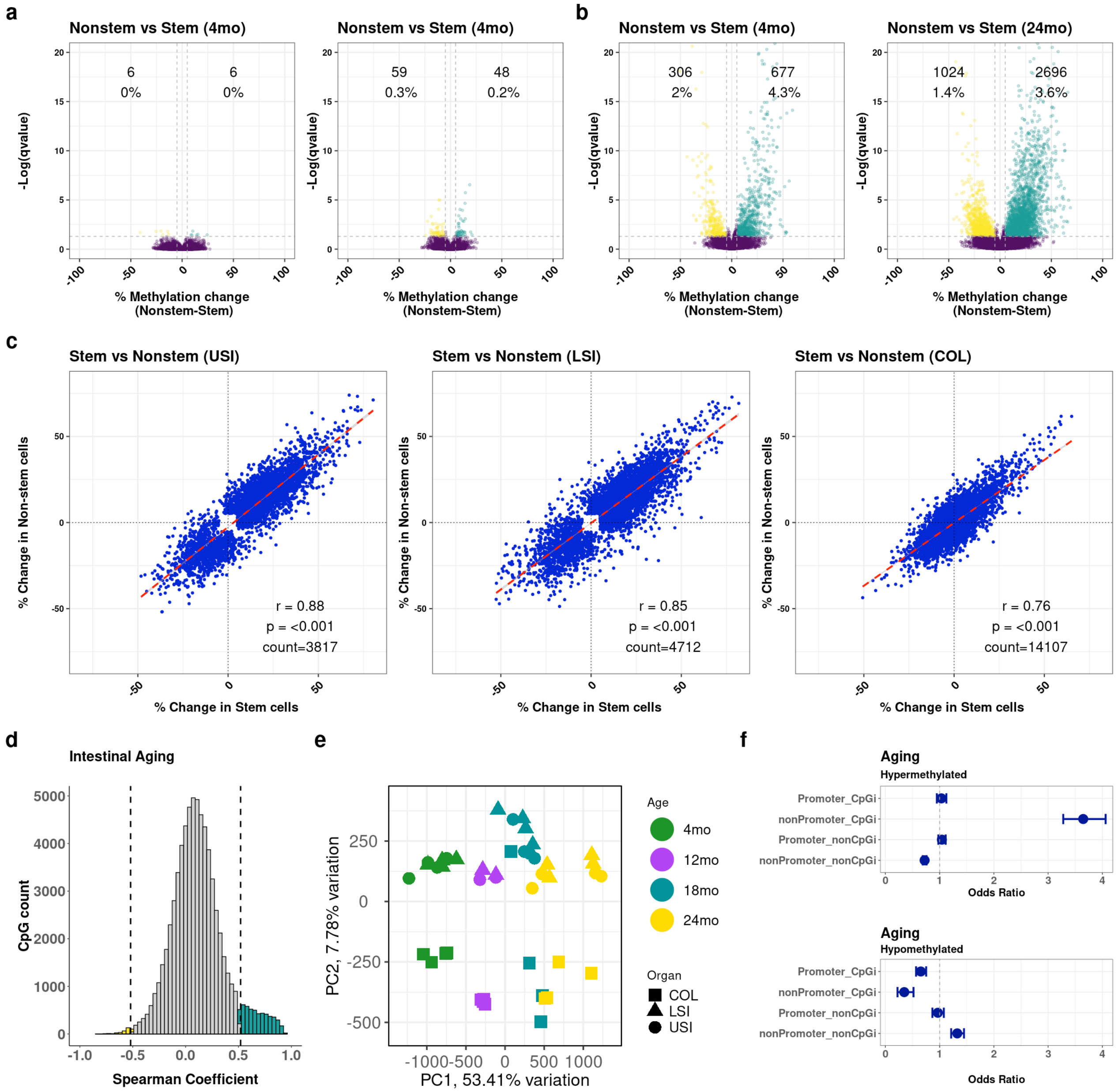

g. Change in differential methylation results between coverage 20 and 30:

	Coverage 20		Coverage 30	
	Hypermethylation	Hypomethylation	Hypermethylation	Hypomethylation
Stem vs Non-stem (4 month)	0%	0%	0%	0%
Stem vs Non-stem (4 month)	0.2%	0.2%	0.2%	0.2%
24-month vs 4-month (stem cells)	3%	1.1%	4.3%	2%
24-month vs 4-month (Non-stem cells)	2.8%	0.9%	3.6%	1.4%

h. Change in correlation coefficient between coverage 20 and 30:

	Coverage 20 (Spearman coefficient)	Coverage 30 (Spearman coefficient)
Stem vs. Non-Stem (USI)	r = 0.86	r = 0.88
Stem vs. Non-Stem (LSI)	r = 0.84	r = 0.85
Stem vs. Non-Stem (COL)	r = 0.86	r = 0.91

Fig. S4

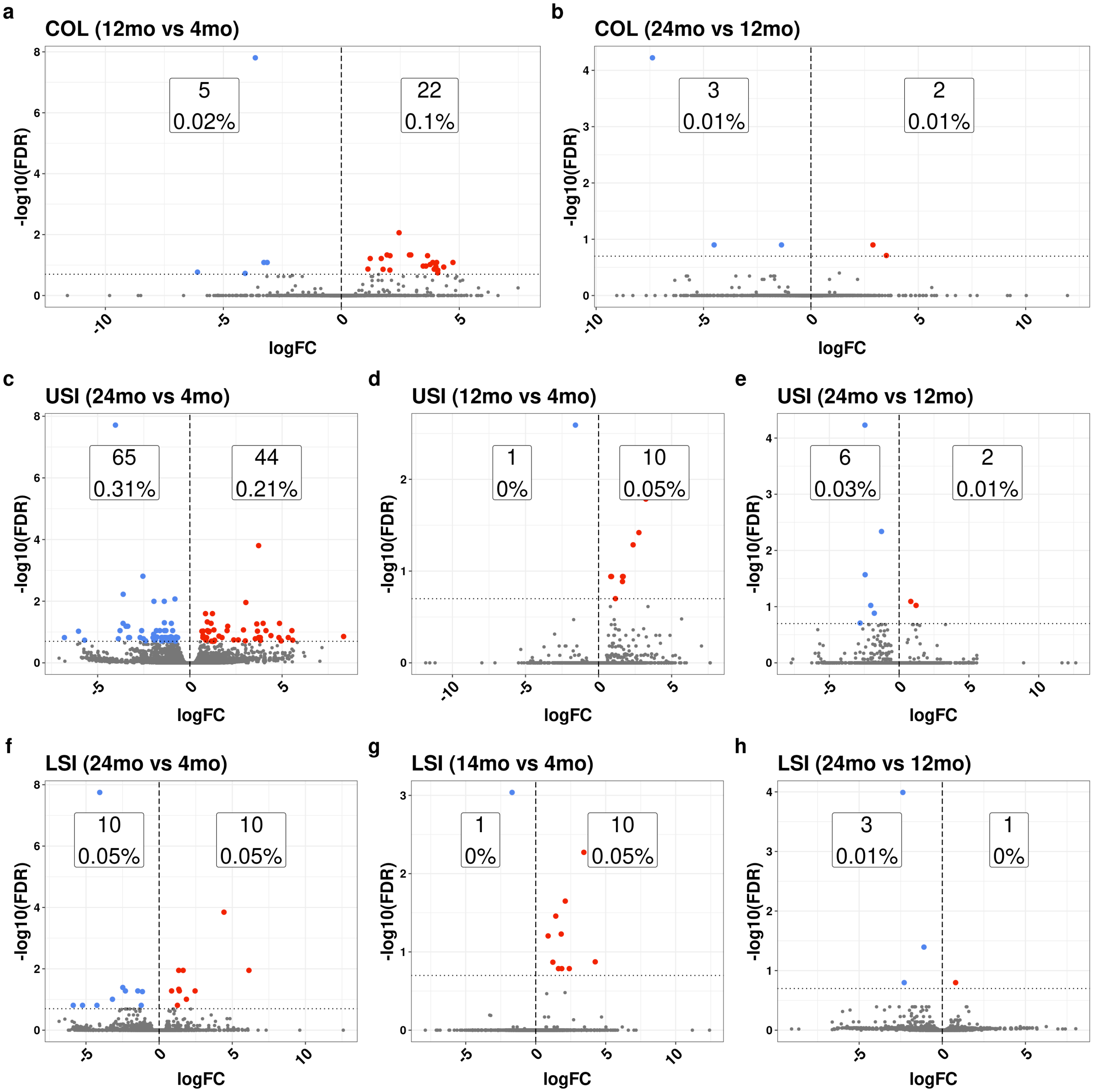

a
COL (12mo vs 4mo)
5
0.02%
22
0.1%
-log10(FDR)
logFC
b
COL (24mo vs 12mo)
3
0.01%
2
0.01%
-log10(FDR)
logFC
c
USI (24mo vs 4mo)
65
0.31%
44
0.21%
-log10(FDR)
logFC
d
USI (12mo vs 4mo)
1
0%
10
0.05%
-log10(FDR)
logFC
e
USI (24mo vs 12mo)
6
0.03%
2
0.01%
-log10(FDR)
logFC
f
LSI (24mo vs 4mo)
10
0.05%
10
0.05%
-log10(FDR)
logFC
g
LSI (14mo vs 4mo)
1
0%
10
0.05%
-log10(FDR)
logFC
h
LSI (24mo vs 12mo)
3
0.01%
1
0%
-log10(FDR)
logFC

Fig. S5

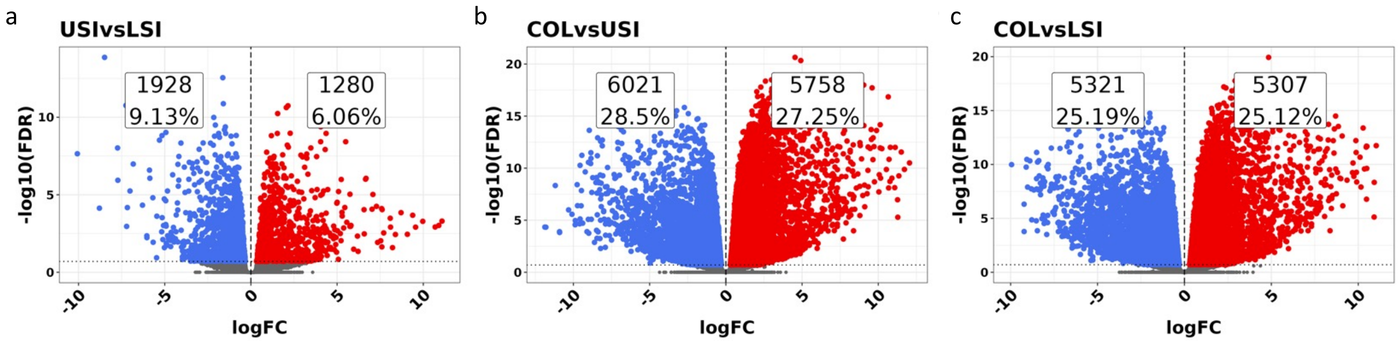
a
USIvsLSI
1928
9.13%
1280
6.06%
-log10(FDR)
logFC
b
COLvsUSI
6021
28.5%
5758
27.25%
-log10(FDR)
logFC
c
COLvsLSI
5321
25.19%
5307
25.12%
-log10(FDR)
logFC

Fig. S6

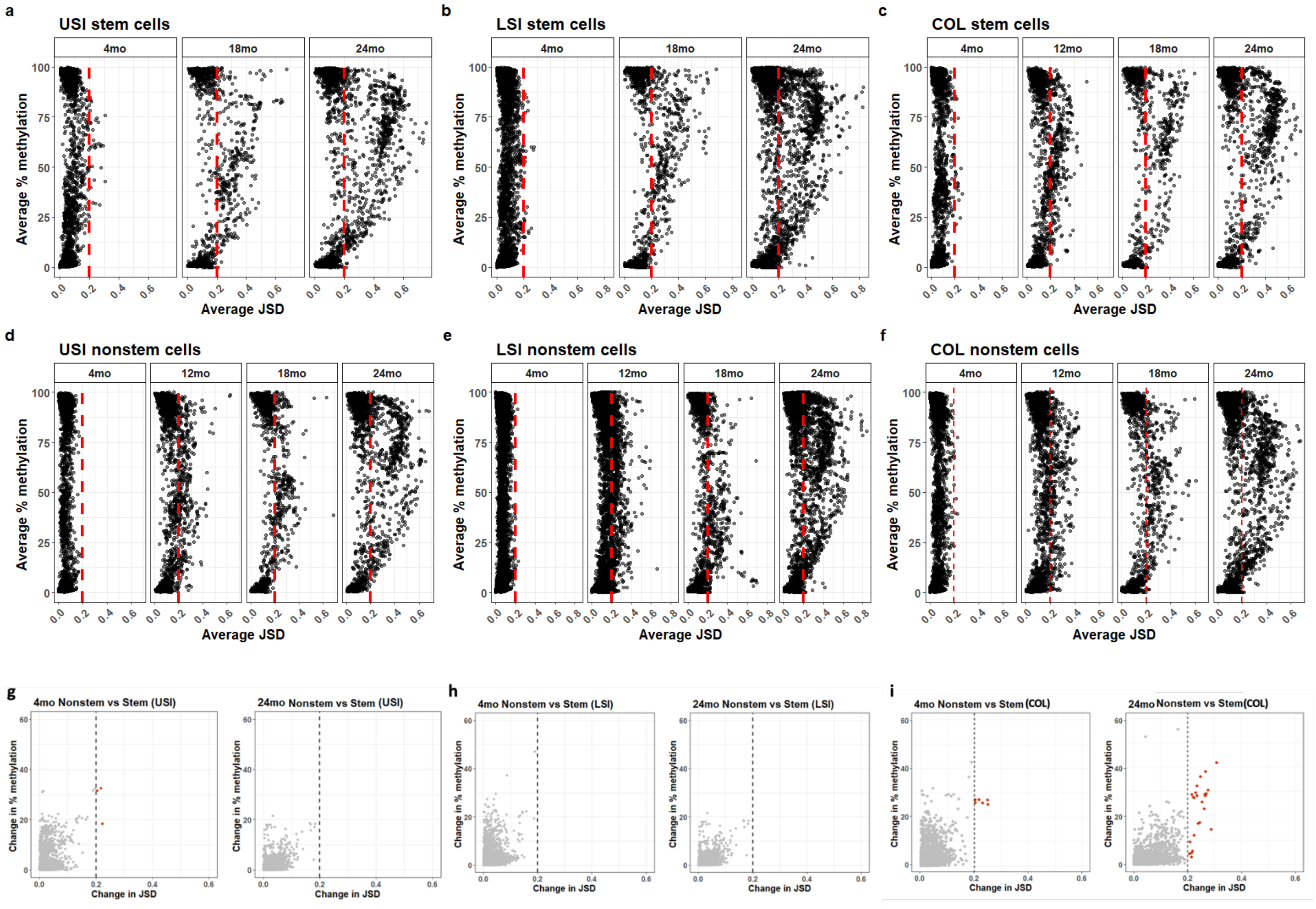
a
USI stem cells
b
LSI stem cells
c
COL stem cells
4mo
12mo
18mo
24mo
Average % methylation
Average JSD
d
USI nonstem cells
e
LSI nonstem cells
f
COL nonstem cells
g
4mo Nonstem vs Stem (USI)
24mo Nonstem vs Stem (USI)
h
4mo Nonstem vs Stem (LSI)
24mo Nonstem vs Stem (LSI)
i
4mo Nonstem vs Stem (COL)
24mo Nonstem vs Stem (COL)
Change in % methylation
Change in JSD

Fig. S7

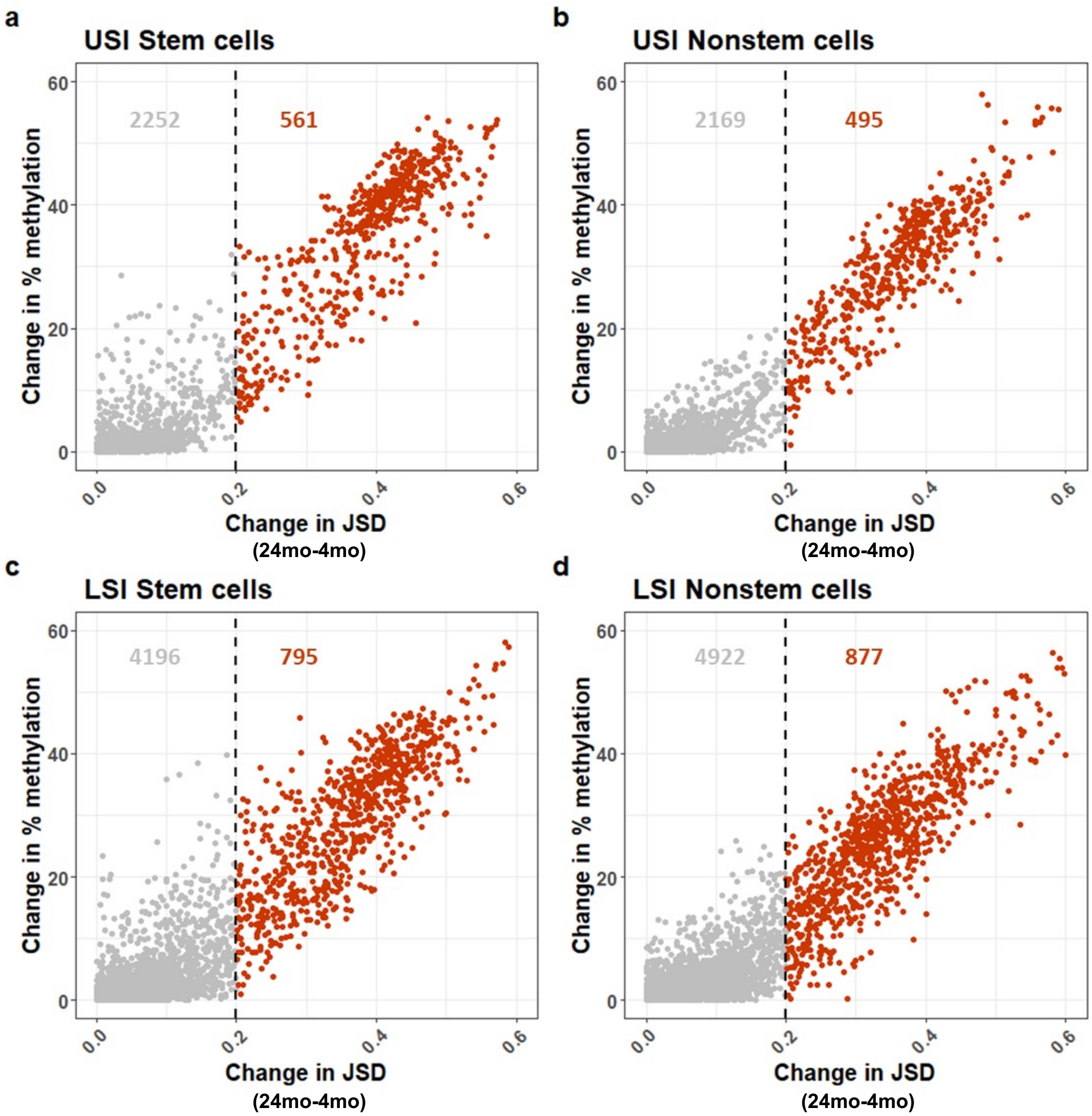
a
USI Stem cells
2252
561
Change in % methylation
Change in JSD
(24mo-4mo)
b
USI Nonstem cells
2169
495
Change in % methylation
Change in JSD
(24mo-4mo)
c
LSI Stem cells
4196
795
Change in % methylation
Change in JSD
(24mo-4mo)
d
LSI Nonstem cells
4922
877
Change in % methylation
Change in JSD
(24mo-4mo)

Fig. S8

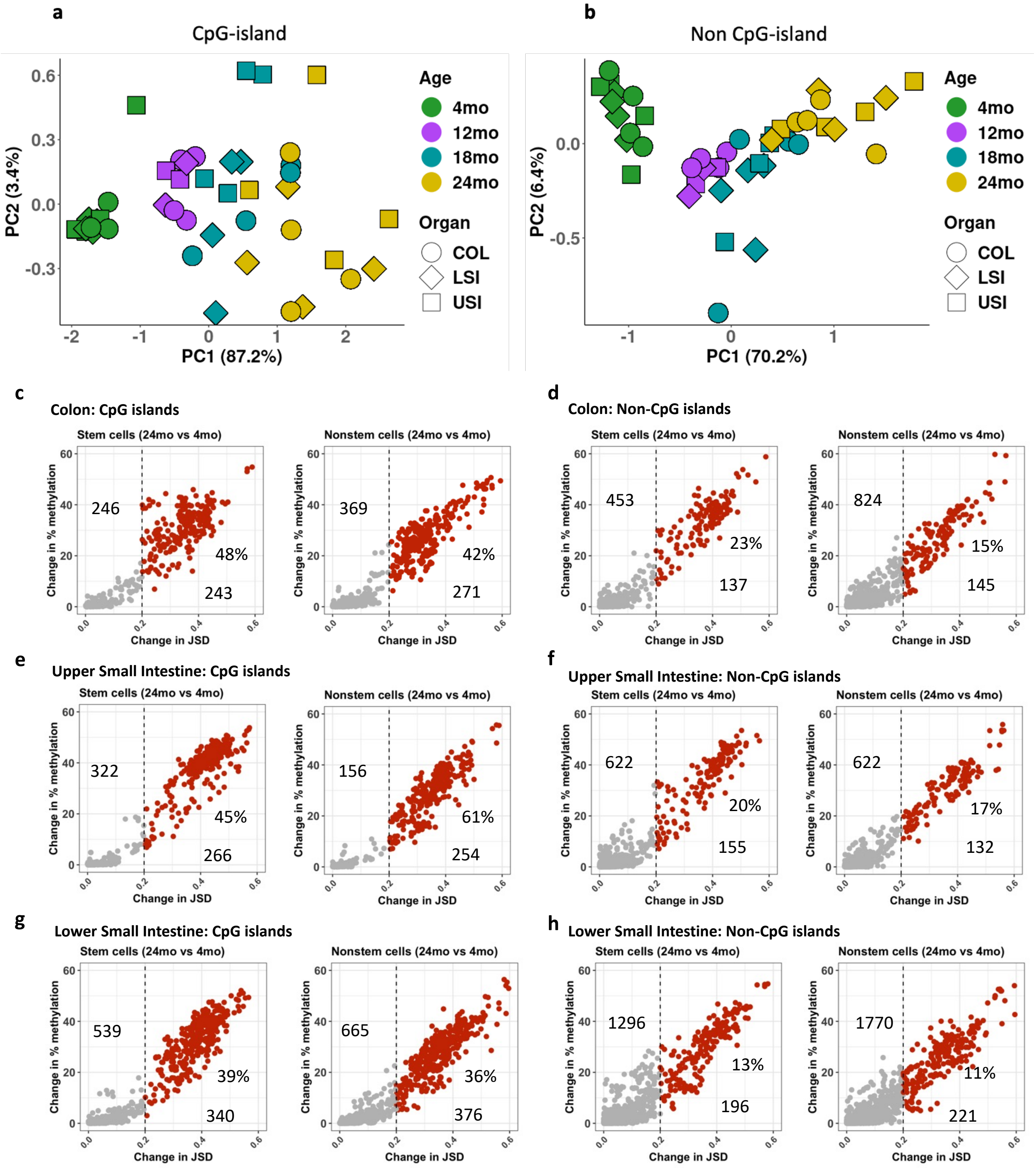
a
CpG-island
b
Non CpG-island
PC2 (3.4%)
PC1 (87.2%)
PC2 (6.4%)
PC1 (70.2%)
Age
4mo
12mo
18mo
24mo
Organ
COL
LSI
USI
c
Colon: CpG islands
Stem cells (24mo vs 4mo)
Nonstem cells (24mo vs 4mo)
246
48%
243
369
42%
271
d
Colon: Non-CpG islands
453
23%
137
824
15%
145
e
Upper Small Intestine: CpG islands
322
45%
266
156
61%
254
f
Upper Small Intestine: Non-CpG islands
622
20%
155
622
17%
132
g
Lower Small Intestine: CpG islands
539
39%
340
665
36%
376
h
Lower Small Intestine: Non-CpG islands
1296
13%
196
1770
11%
221
Change in % methylation
Change in JSD

Fig. S9

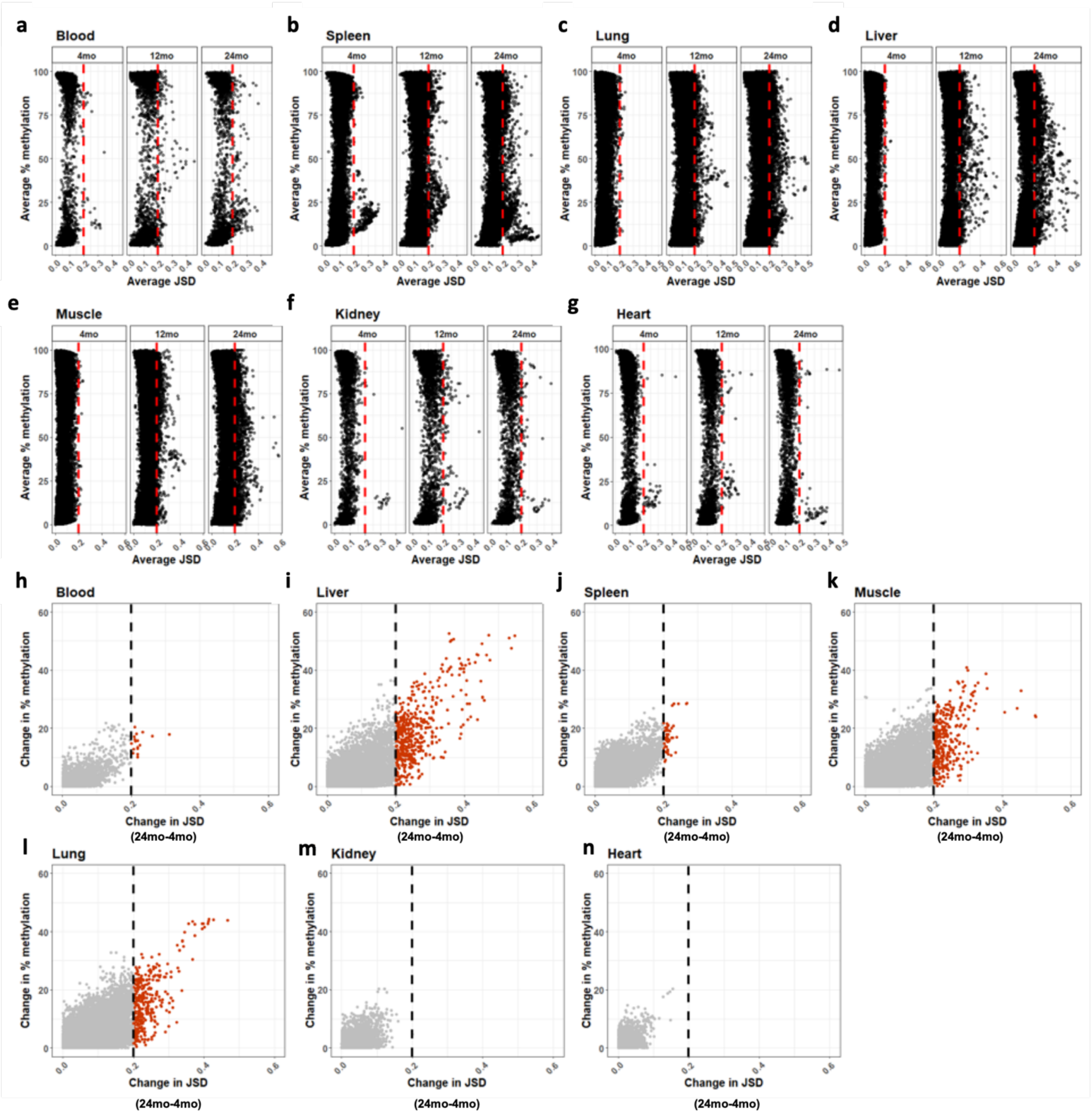
a
Blood
b
Spleen
c
Lung
d
Liver
e
Muscle
f
Kidney
g
Heart
4mo
12mo
24mo
Average % methylation
Average JSD
h
Blood
i
Liver
j
Spleen
k
Muscle
l
Lung
m
Kidney
n
Heart
Change in % methylation
Change in JSD
(24mo-4mo)

Fig. S10

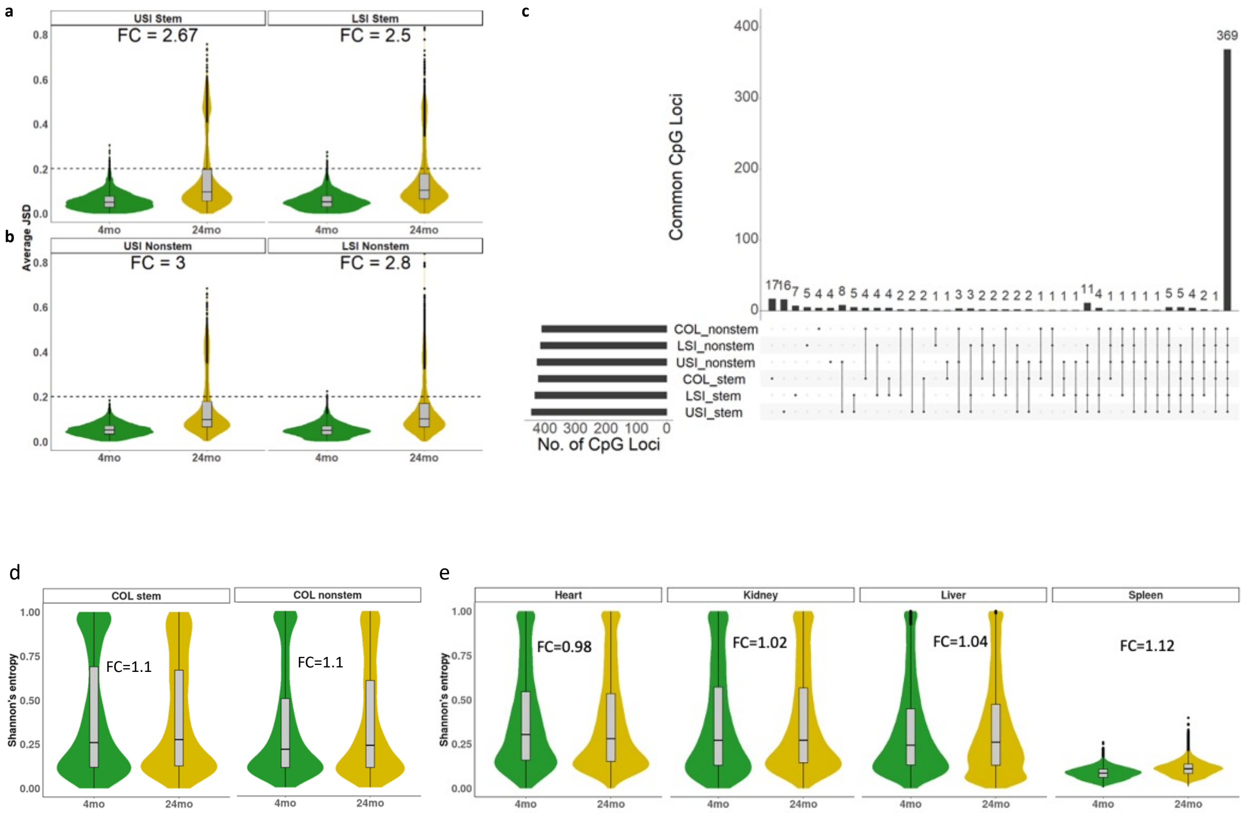
a
USI Stem
LSI Stem
FC = 2.67
FC = 2.5
b
USI Nonstem
LSI Nonstem
FC = 3
FC = 2.8
Average JSD
4mo
24mo
c
Common CpG Loci
369
COL_nonstem
LSI_nonstem
USI_nonstem
COL_stem
LSI_stem
USI_stem
No. of CpG Loci
d
COL stem
COL nonstem
FC=1.1
FC=1.1
Shannon's entropy
e
Heart
Kidney
Liver
Spleen
FC=0.98
FC=1.02
FC=1.04
FC=1.12

Fig. S1: DNA methylation change with differentiation in the small intestine. **a-d**. Volcano plot showing methylation differences between stem cell (Lgr5-GFP+) and nonstem cells (Lgr5-GFP-) in young (4 month) upper small intestine (a), young (4 month) lower small intestine (b), old (24 month) upper small intestine (c), old (24 month) lower small intestine (d). DNA methylation change with age in the small intestine. **e-f**. Volcano plot showing methylation differences between old (24 month) and young (4 month) Lgr5-GFP+ stem cells in upper small intestine (e), lower small intestine (f), between old (24 month) and young (4 month) Lgr5-GFP- nonstem cells in upper small intestine (g), lower small intestine(h). CpG sites that change with age in different intestinal tissues. **i-k**. Scatter plot of CpG sites that change with age compared between stem cells (Lgr5-GFP+) from different pairs of tissues (**i**) upper small intestine (USI) and lower small intestine (LSI), (**j**) upper small intestine (USI) and colon (COL), (**k**) lower small intestine (LSI) and colon (COL). **l-n** Scatter plot of CpG sites that change with age compared between nonstem cells (Lgr5-GFP-) from different pairs of tissues (**l**) upper small intestine (USI) and lower small intestine (LSI), (**m**) upper small intestine (USI) and colon (COL), (**n**) lower small intestine (LSI) and colon (COL).

Fig. S2: Differential methylation and permutation analysis at coverage 30**. a**. Volcano plots showing methylation differences between stem cells (Lgr5-GFP+) and nonstem cells (Lgr5-GFP-) in young (left) and old (right) mouse colon samples. Each volcano plot indicates the number and percent of CpG sites that are differentially methylated (Methylation change >±5%, q-value <0.05). **b.** Volcano plots showing methylation differences between young (4 month) and old (24 month) samples in stem cells and nonstem cells. **c**. Scatterplot of CpG sites that change significantly between 4-month versus 24 month in either stem cells, nonstem cells or both in COL, USL and LSI. **d**. Histogram of Spearman correlation coefficients (r) derived from permutation analysis of 70,541 CpG sites. Significantly (empirical p-value<0.05, r >|0.5|) hypermethylated CpG sites are in red and hypomethylated sites are in green. **e**. PCA plot constructed using the 5,503 CpG sites from the permutation test that significantly change with age. **f**. Odds ratios that CpG sites in given genomic regions (Promoter-CpGi, nonPromoter-CpGi, Promoter-nonCpGi, nonPromoter-nonCpGi) are more likely to gain methylation with age (top) or lose methylation with age (bottom). **g**. Table comparing change in differential methylation results when coverage is 20 versus 30. **h**.

Table comparing correlation coefficients with change with age in stem cells versus nonstem cells when coverage is 20 versus 30.

Fig. S3: Distribution of spearman r-values and PCA of significant CpG sites. (**a**) Histograms of observed (magenta) and simulated (green) r-values between age and methylation. (**b**) PCA analysis of significantly hypermethylated aging sites in nonPromoter-CpGi region (1139 CpG sites).

Fig. S4: Changes in gene expression with age different intestinal tissue sections. **a**-**b** Volcano plot showing differential gene expression in colon (COL) samples between (**a**) 12-month vs 4 month, (**b**) 24-month vs 12-month. **c**-**e**. Volcano plot showing differential gene expression in upper small intestine (USI) samples between (**c**) 24-month vs 4 month, (**d**) 12-month vs 4 month, (**e**) 24-month vs 12 month. **f**-**h**. Volcano plot showing differential gene expression in lower small intestine (LSI) samples between (**f**) 24-month vs 4 month, (**g**) 12-month vs 4 month, (**h**) 24-month vs 12 month.

Fig. S5: Changes in gene expression within different intestinal tissue sections. Volcano plot showing differential gene expression in intestinal tissue in (**a**) upper small intestine (USI) vs lower small intestine (LSI), (**b**) upper small intestine (USI) vs colon (COL) and (**c**) upper small intestine (USI) vs colon (COL).

Fig. S6: Jensen-Shannon Distribution (JSD) across Intestinal tissues in stem and nonstem cells in 4-12-18-24-month samples. **a-c**. Scatterplot of Jensen Shannon distribution values with age (4-month, 12-month, 18 month and 24 month) across stem cells (Lgr5-GFP+) in (**a**) upper small intestine (USI), (**b**) lower small intestine (LSI) and (**c**) Colon. **d-f**. Scatterplot of Jensen Shannon distribution values with age (4-month, 12-month, 18-month and 24-month) across nonstem cells (Lgr5-GFP-) in (**d**) upper small intestine (USI), (**e**) lower small intestine (LSI) and (**f**) Colon. Entropy changes between stem and nonstem cells in the small intestine and colon. Scatterplots showing the change in Jensen-Shannon Distance

(JSD) on the x-axis vs change in methylation on y-axis, between (left) young and old stem cells (Lgr5-GFP+), and (right) young and old nonstem cells (Lgr5-GFP-) in (**g**) Upper small intestine (USI), (**h**) Lower small intestine (LSI) and (**i**) colon (COL).

Fig. S7: Entropy increases with age in the small intestine*.* Scatterplots showing the change in Jensen-Shannon Distance (JSD) on the x-axis vs change in methylation on y-axis in (**a-b**) Upper small intestine (USI) and (**c-d**) Lower small intestine (LSI) between (left) young and old stem cells (Lgr5-GFP+), and (right) young and old nonstem cells (Lgr5-GFP-).

Fig. S8: Change in Jensen Shannon distribution (JSD) values between old and young in across different organ samples**. a**-**g**. Scatterplot of Jensen Shannon distribution values with age (4-month, 12 month, and 24 month) in different tissue samples (**a**) Whole blood, (**b**) Spleen, (**c**) Lung, (**d**) Liver, (**e**) Skeletal muscle, (**f**) Kidney and (**g**) Heart. **h**-**n**. Scatterplots showing the change in Jensen-Shannon Distance (JSD) on the x-axis vs change in methylation on y-axis between young (4 month) and old (24 month) samples in the (**h**) Whole blood, (**i**) Spleen, (**j**) Lung, (**k**) Liver, (**l**) Skeletal muscle, (**m**) Kidney and (**n**) Heart.

Fig. S9: Change in entropy in CpG islands vs nonCpG islands. (**a-b**) PCA plots constructed using Jensen-Shannon Distances (JSDs) for loci with coverage > 40 in CpG islands (**a**) and nonCpG islands (**b**). Scatterplots showing the change in Jensen-Shannon Distance (JSD) on the x-axis vs change in methylation on y-axis in between stem cells and nonstem cells in small intestine and colon in CpG islands (left) vs non CpG islands(right). (**c-d**) Colon, (**e-f**) Upper small intestine and (**g-h**) Lower small intestine.

Fig. S10: Change in Entropy in different parts of the intestine. (**a**) Average Jensen Shannon Distances for each loci (JSD) in young (4 month) and old (24 month) mice in stem (Lgr5-GFP+) cells in Upper small intestine (USI) and in Lower small intestine (LSI). (**b**) Same as in a in nonstem (Lgr5-GFP-) cells. (**c**) UpSet plots of shared/unique loci with Jensen Shannon distance (JSD) >0.2 in stem cells and nonstem cells of different sections of the intestine USI (upper small intestine), LSI (lower small intestine) and COL

(Colon). (**d**) Average Shannon’s entropy measures for colon stem cells and nonstem cells in young (4 month) and old (24 month) mice. (**e**) Average Shannon’s entropy measures for heart, kidney, liver and spleen in young (4 month) and old (24 month) mice.

Supplementary Method

Jensen Shannon Distance (JSD) is not the current standard method to quantify entropy in the field, and it differs from Shannon's entropy, which could be creating some confusion. We would like to clarify that Jensen Shannon Distance (JSD) is a method of calculating entropy with a distance metric using a reference sample. If the reference sample and test sample distributions don't overlap at all then JSD equals 1(which is the maximum), if they overlap completely then JSD equals 0, and with intermediate levels of overlap the value ranges between 0 and 1.

Example 1: Case of low JSD change between test sample and reference distribution

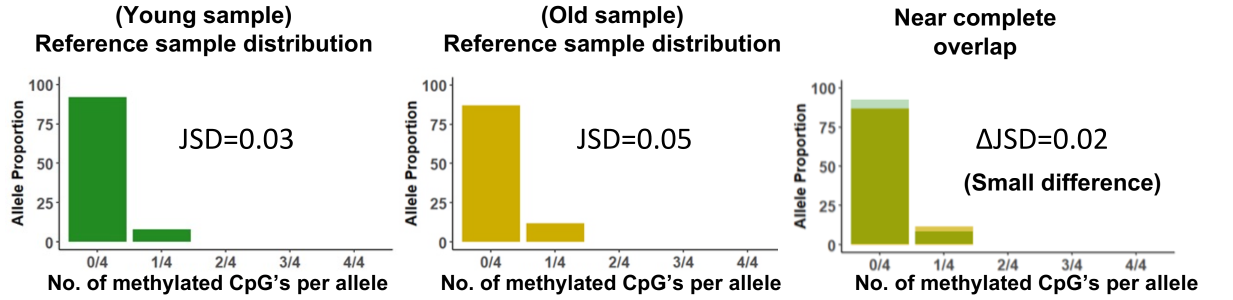

Histogram shows the allele distribution on the X-axis and the proportion of each allele in the y-axis.

Example 2: Case of high JSD change between test sample and reference distribution

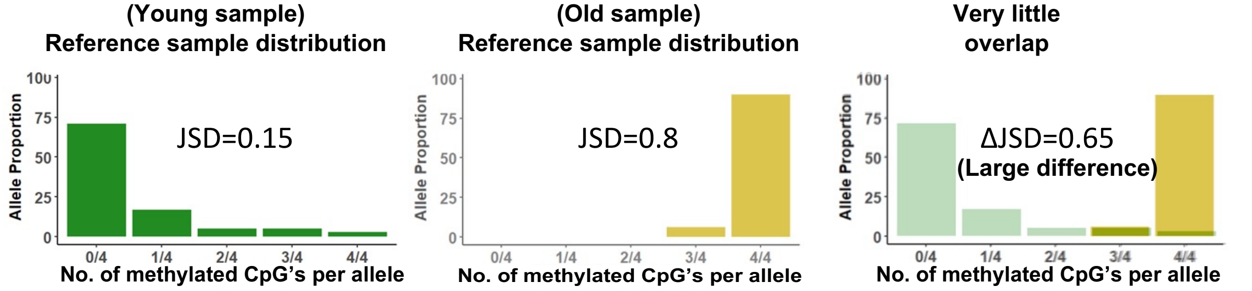

Histogram shows the allele distribution on the X-axis and the proportion of each allele in the y-axis.

Thus, a change in the methylation distribution from the reference sample results in increasing JSD.

<u>Shannon's Entropy</u>

More conventional methods of quantifying entropy such as Shannon's entropy do not consider distribution and direction of methylation change, resulting in the possibility of the alleles having the same number of fully methylated or unmethylated CpG's but a very different distribution. For example (figure below), when alleles from the young sample are unmethylated whereas alleles from the old sample are fully methylated, the value of Shannon's entropy between the samples will be the same but JSD shows a high difference. Therefore, we think JSD gives a better measure of age-related entropy.

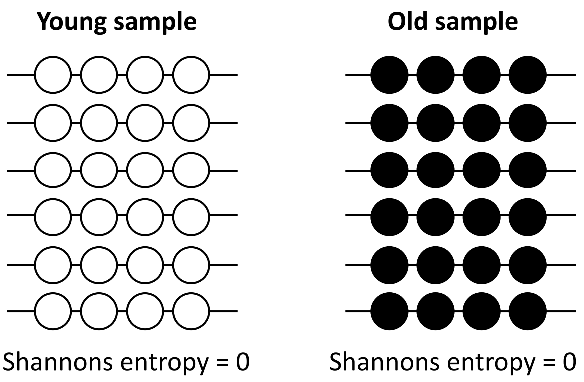

$$\textbf{Entropy(H)} = \sum_{i=1}^{n=X} \frac{-p\log 2(p) - q\log 2(q)}{n}$$

H - Shannon's information entropy

P = Probability of methylated site
Q = Probability of unmethylated site
X = No. of alleles

As an example, we calculated Shannon's entropy for a locus with high change in JSD (example below) between reference and test sample distribution and the change in Shannon's entropy is very small, only 0.08, whereas the change in JSD is quite large at 0.65.

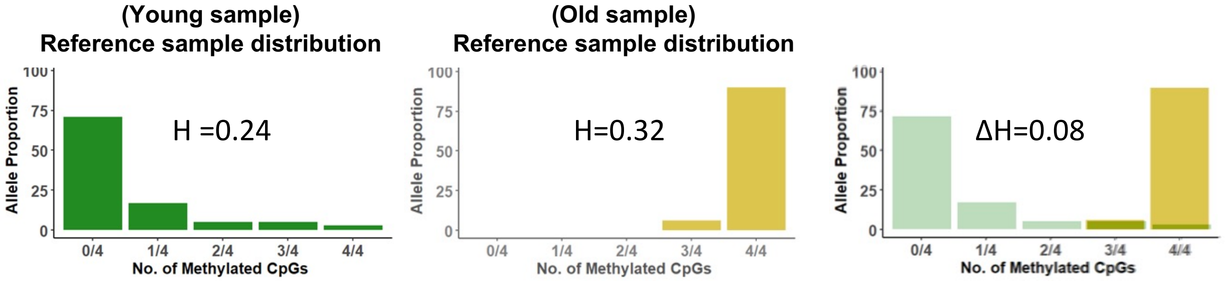

H= Shannon's entropy,
∆H= change Shannon's entropy

Histogram shows the allele distribution on the X-axis and the proportion of each allele in the y-axis.

More details on this are in the following reference papers :

<u>Jensen Shannon Distance:</u>
Jenkinson G, Abante J, Feinberg AP, Goutsias J. An information-theoretic approach to the modeling and analysis of whole-genome bisulfite sequencing data. BMC Bioinformatics. 2018;19(1)

<u>Shannon's entropy:</u>
Landan G, Cohen NM, Mukamel Z, Bar A, Molchadsky A, Brosh R, et al. Epigenetic polymorphism and the stochastic formation of differentially methylated regions in normal and cancerous tissues. Nat Genet. 2012;44(11):1207-14
