# Supplementary material for: DNA methylation entropy as a measure of stem cell replication and aging
Source: Genome Biol. 2023 Feb 16;24:27. doi: 10.1186/s13059-023-02866-4 (PMC9933260; doi:10.1186/s13059-023-02866-4)
Supplement: Supplementary file 2 — Additional file 2: Contains all supplementary tables. [file 13059_2023_2866_MOESM2_ESM.pdf]

Table S1

DNA methylation change between stem and nonstem cells in small intestine and colon in different genomic compartments

Tissue Section	Genomic region	No. of sites	Hypermethylated sites	Hypomethylated sites
Colon (4 months) (Nonstem-Stem)	CpGi-Promoters (pCGI)	5780	0 (0%)	0 (0%)
	CpGi-nonPromoters (npCGI)	2354	0 (0%)	0 (0%)
	nonCpGi-Promoters (pnCGI)	8453	0 (0%)	0 (0%)
	nonCpGi-nonPromoters (npnCGI)	73926	2 (0%)	4 (0%)
Colon (24 months) (Nonstem-Stem)	CpGi-Promoters (pCGI)	1599	0 (0%)	0 (0%)
	CpGi-nonPromoters (npCGI)	775	0 (0%)	0 (0%)
	nonCpGi-Promoters (pnCGI)	3128	7 (0.2%)	7 (0.2%)
	nonCpGi-nonPromoters (npnCGI)	33236	61 (0.2%)	65 (0.2%)
USI (4 months) (Nonstem-Stem)	CpGi-Promoters (pCGI)	10997	19 (0.1%)	0 (0%)
	CpGi-nonPromoters (npCGI)	3967	7 (0.2%)	0 (0%)
	nonCpGi-Promoters (pnCGI)	15954	86 (0.5%)	3 (0%)
	nonCpGi-nonPromoters (npnCGI)	105586	751 (0.7%)	20 (0%)
USI (24 months) (Nonstem-Stem)	CpGi-Promoters (pCGI)	10600	32 (0.3%)	1 (0%)
	CpGi-nonPromoters (npCGI)	4462	44 (1%)	8 (0.2%)
	nonCpGi-Promoters (pnCGI)	13554	264 (1.9%)	32 (0.2%)
	nonCpGi-nonPromoters (npnCGI)	123165	2411 (2%)	432 (0.3%)
LSI (4 months) (Nonstem-Stem)	CpGi-Promoters (pCGI)	19122	2 (0%)	0 (0%)
	CpGi-nonPromoters (npCGI)	7607	6 (0.1%)	0 (0%)
	nonCpGi-Promoters (pnCGI)	23319	9 (0%)	1 (0%)
	nonCpGi-nonPromoters (npnCGI)	206826	112 (0%)	9 (0%)
LSI (24 months) (Nonstem-Stem)	CpGi-Promoters (pCGI)	5666	2 (0%)	0 ()%)
	CpGi-nonPromoters (npCGI)	2911	24 (0.8%)	3 (0.1%)
	nonCpGi-Promoters (pnCGI)	9488	48 (0.5%)	21 (0.2%)
	nonCpGi-nonPromoters (npnCGI)	102248	578 (0.6%)	272 (0.3%)

Table S2

DNA methylation change with age in small intestine and colon in different genomic compartments

Tissue Section	Genomic region	No. of CpG sites	Hypermethylated sites	Hypomethylated sites
Colon (Stem Cells) (Old-Young)	CpGi-Promoters (pCGI)	1396	140 (10.%)	1 (0.1%)
	CpGi-nonPromoters (npCGI)	694	204 (29.4%)	0 (0%)
	nonCpGi-Promoters (pnCGI)	2607	151 (5.9%)	33 (1.3%)
	nonCpGi-nonPromoters (npnCGI)	27343	890 (3.2%)	454 (1.6%)
Colon (Nonstem Cells) (Old-Young)	CpGi-Promoters (pCGI)	9713	673 (6.9%)	5 (0%)
	CpGi-nonPromoters (npCGI)	3944	612 (15.2%)	0 (0%)
	nonCpGi-Promoters (pnCGI)	12518	637 (5.1%)	87 (0.7%)
	nonCpGi-nonPromoters (npnCGI)	108082	2160 (2%)	1244 (1.1%)
USI (Stem Cells) (Old-Young)	CpGi-Promoters (pCGI)	14383	649 (4.5%)	12 (0.1%)
	CpGi-nonPromoters (npCGI)	4375	495 (11.3%)	57 (0.4%)
	nonCpGi-Promoters (pnCGI)	15263	719 (4.7%)	57 (0.4%)
	nonCpGi-nonPromoters (npnCGI)	94971	2871 (3%)	832 (0.9%)
USI (Nonstem Cells) (Old-Young)	CpGi-Promoters (pCGI)	8845	272 (3.1%)	1 (0%)
	CpGi-nonPromoters (npCGI)	4259	511 (12 %)	1 (0%)
	nonCpGi-Promoters (pnCGI)	14670	455 (3.1%)	114 (0.8%)
	nonCpGi-nonPromoters (npnCGI)	146389	2182 (1.5%)	1341 (1%)
LSI (Stem Cells) (Old-Young)	CpGi-Promoters (pCGI)	7520	488 (6.5%)	2 (0%)
	CpGi-nonPromoters (npCGI)	3429	576 (16.8%)	0 (0%)
	nonCpGi-Promoters (pnCGI)	12474	671 (5.4%)	97 (0.8%)
	nonCpGi-nonPromoters (npnCGI)	128638	3765 (2.9%)	1291 (1%)
LSI (Nonstem Cells) (Old-Young)	CpGi-Promoters (pCGI)	9593	614 (6.4%)	1 (0%)
	CpGi-nonPromoters (npCGI)	4421	738 (16.7%)	124 (0.9%)
	nonCpGi-Promoters (pnCGI)	13491	584 (4.3%)	124 (0.9%)
	nonCpGi-nonPromoters (npnCGI)	130267	2481 (2%)	1202 (1%)

Table S3

30-day turnover rate per tissue

Organ/Tissue	Turnover time	No. of turnover /30 days	Method	Reference
Small intestine	2-3 days	10	3H-thymidine	Potten [33]
Colon	3-4 days	8.5	3H-thymidine	Potten [33]
Blood (Hematopoietic Stem cells)	57 days	0.52	Bromodeoxyuridine	Chesier [34]
Liver Hepatocytes	200-400 days	0.1	3H-thymidine	Magami et. al [35]
Lung	Bronchial epithelium (2-7 days)<br>Bronchiolar epithelium- (10 days)<br>Alveolar epithelium- (28-35 days)<br><br>Average =10.5 days	3.53	3H-thymidine	Bowden [36]
Straited muscle (data only in rat muscle)	>170 days	0.17	3H-thymidine	Allbrook et. al [37]
Spleen (whole)	9 days	3.3	Bromodeoxyuridine	Crippen et. al [38]
Kidney (renal corpuscle and the nephron tubule)	>120 days	0.25	3H-thymidine	Litvak & Baserga [39]
Cardiomyocytes	>300 days	0.1	3H-thymidine	Cluzeaut & Maurer-Schultze [40]

Table S4

Sample pooling information

Tissue	Age	Number of Biological replicates	Number of animals per pool
USI (both stem/non-stem)	4-month	2 (each pooled)	4
LSI (both stem/non-stem)	4-month	2 (each pooled)	4
Colon (both stem/non-stem)	4-month	2 (each pooled)	4
USI (both stem/non-stem)	12-month	2 (each pooled)	3
LSI (both stem/non-stem)	12-month	2 (each pooled)	3
Colon (both stem/non-stem)	12-month	2 (each pooled)	3
USI (both stem/non-stem)	18-month	2 (each pooled)	2
LSI (both stem/non-stem)	18-month	2 (each pooled)	2
Colon (both stem/non-stem)	18-month	2 (each pooled)	2
USI (both stem/non-stem)	24-month	2 (each pooled)	2
LSI (both stem/non-stem)	24-month	2 (each pooled)	2
Colon (both stem/non-stem)	24-month	2 (each pooled)	2
Whole Blood	4-month	3	NA
Whole Blood	12-month	3	NA
Whole Blood	24-month	4	NA
Heart	4-month	6	NA
Heart	12-month	6	NA
Heart	24-month	10	NA
Kidney	4-month	3	NA
Kidney	12-month	3	NA
Kidney	24-month	4	NA
Liver	4-month	2	NA
Liver	12-month	2	NA
Liver	24-month	2	NA
Lung	4-month	2	NA
Lung	12-month	2	NA
Lung	24-month	2	NA
Skeletal Muscle	4-month	2	NA
Skeletal Muscle	12-month	2	NA
Skeletal Muscle	24-month	2	NA
Spleen	4-month	2	NA
Spleen	12-month	2	NA
Spleen	24-month	2	NA
