# Supplementary material for: DNA methylation entropy as a measure of stem cell replication and aging
Source: Genome Biol. 2023 Feb 16;24:27. doi: 10.1186/s13059-023-02866-4 (PMC9933260; doi:10.1186/s13059-023-02866-4)
Supplement: Supplementary file 3 — Additional file 3: Review history. [file 13059_2023_2866_MOESM3_ESM.docx]

**1^st^ round**

**Reviewer 1**

Vaidya et al. have analysed DNA methylation (DNAm) ageing-related changes in stem and non-stem mouse intestinal cells to test whether ageing-related DNAm drift is linked with stem cell division. The mechanism of stochastic DNAm drift is not yet precisely defined, and as well as general ageing analysis, they quantified entropy to explore this further.

Mice across the age range of 4, 12, 18, or 24 months were evaluated. FACS sorting was used to isolate stem cell and differentiated cells and 3 components of the tract were examined - upper (USI) & lower (LSI) small intestine, and colon (COL).

DNAm analysis was via RRBS, with a minimum coverage of 20 read per CpG and a total of 180,496 CpG sites were detected across all samples. The authors identify by PCA that tissue-type was a more significant driver of variation than age. In initial analysis between the two timepoints of old and young, DNAm age-changes in both stem and non-stem cells were found to be highly correlated and were also seen to occur in the same CpGs in the 3 different components of the intestine.

In an analysis across all 4 time-points (in 125,077 CpGs), significant ageing-CpGs, which were assessed by permutation, identified a strong hypermethylation signature. Non-promoter CpG islands were found to have 3.5x odds of hypermethylating with age. There were very few CpGs that were found to be common between ageing and tissue-related changes, and, thus, the authors concluded that these are distinct biological processes.

RNA-seq analysis in these same sets clustered by tissue, not age, and with few genes showing changes between the two age extremes. Overall, DNAm ageing changes were found to be broader than gene expression changes.

Entropy was quantified by Jensen-Shannon Distance (JSD) in 10,366 loci (4 CpGs within 60 bp with minimum coverage 40 reads) detectable across all samples. These results clustered solely on age, with a strong correlation between old stem and old non-stem cells. Finally, the authors compared tissues with highest (intestine), high (liver, lung, spleen) versus low (heart, kidney, skeletal) stem cell division and identified ageing related DNAm entropy is principally an effect of stem cell division and highly tissue specific.

The paper's findings are interesting and will be of significance to the ageing and epigenomics community. My comments for the authors are listed below.

Major

1. With the multiple analysis and sub-analyses, the manuscript becomes difficult to follow for the reader at times. Would benefit from the authors re-assessing readability with a fresh eye.

2. The threshold for RRBS was an appropriate minimum coverage level of 20 reads at each CpG, however, concordance increases with depths >30 [1] - could the authors explore whether higher reads depths show consistency with a representative fraction of their current results? Particularly the effect of only requiring at least 75% of the samples to reach 20 read minimum of the CpGs included in the 4-age group analysis?

3. It is stated 'significant with age (p-value < 0.05)' on line 90 - is this corrected or nominal?

4. Regarding the RNA-seq analysis and the non-promoter CpG island hypermethylation - was there any evidence of changes in expression of non-canonical isoforms with age?

5. Brief explanation should be included in the Results of what comprises the definition of the 10,366 loci in the Entropy results - so that the reader does not need to go trawling through methods to understand this.

6. Regarding the DNAm entropy calculated loci - how is this impacted on by CpG density - i.e., is the ageing signal being driven by dense or sparse CpG loci?

7. This is not experimentally required for this paper - but authors should acknowledge in the Discussion that it would clearly be of significant interest if a consistent result was found with the now available Illumina mouse DNAm array - which avoids all the vagaries of stochastic sequencing depth variation.

Minor

1. Spelling 'og' line 139

2. Spelling 'pattens' line 195

1. Sun, X., et al., A comprehensive evaluation of alignment software for reduced representation bisulfite sequencing data. Bioinformatics, 2018. 34(16): p. 2715-2723.

**Reviewer 2**

Vaidya et al. follow their hypothesis that age-associated DNA methylation is a process of methylation drift that is linked to stem cell division. They suggest that while DNA methylation overall is primarily tissue-specific, DNA methylation entropy is exclusively an aging phenomenon. Furthermore, they indicated that most age-related DNA methylation drift should be attributed to adult stem cell replication. While the idea is interesting and relevant, several technical aspects are difficult to follow. Overall, a better validation might be needed to support their statements.

I have the following specific comments:

1) Tissue specific differences were analyzed on epithelial cells from colon and small intestine - these closely related cell types/tissues may not be ideal to generalize for tissue-specific differences. Since the authors also analyzed many other tissues it would be better to consider these for the analysis, too.

2) The number of biological replica should be better indicated in the manuscript. Furthermore, four mouse samples were pooled for RRBS. Is it conceivable that mixing of samples impacts on the JSD if there are interindividual differences?

3) The concept of JSD needs to be much better clarified in the manuscript - particularly since this is the most innovative aspect. Looking at other studies I got the impression that JSD is typically higher at hemimethylated sites (peaking at 50% methylation level) whereas it is generally lower at none or highly methylated sites. If so, this needs to be better clarified and taken into consideration. It is known that age-related CpGs are often hemimethylated, while tissue-specific DNA methylation may be more at the extremes. This might at least contribute to the observed discrepancy in JSD.

4) The reference distribution for calculation of JSD should be better clarified.

5) It is unclear how tissue renewal rates were measured (e.g. in Figure 5c). Blood is a tissue with enormous turnover. I am surprised to find the tissue renewal rate/30% for blood at the lower edge of all tissues.

6) On the other hand, there is a bigger hierarchy in the hematopoietic system between the quiescent long-term repopulating hematopoietic stem cells and the various intermediate faster proliferating progenitor cells. Separation in stem cells and none-stem cells might be too simplistic for many tissues (that even comprise multiple cell types).

7) Colon stem cells in Figure 5 seem to be on the same line as non-stem cells. This might argue against a clear finding toward their hypothesis. Why are renewal rates anticipated similar in stem cells and non-stem cells? Why does Figure 5a only depict COL and not SI?

8) It is well known that even non-proliferative tissues, such as brain, have extensive age-associated DNA methylation. As indicated, this has been shown by many functional signatures. Furthermore, multi-tissue clocks have been successfully established. It therefore appears surprising that their data suggest very little overlap in aging changes between distantly related tissues and between tissues that have very different stem cell proliferation rates.

9) The threefold change was observed in both stem and non-stem cells of 24 months (Figure 5a). How do they conclude that this is due to stem cell division (line 212 as well as line 249-250)? As it stands, there appear to be hardly conclusive results how stem cell division or cell division rate are related to the epigenetic drift.

Minor:

10) The GEO ID has not yet been provided.

11) The conclusion of lines 78-80 is not fully supported by the corresponding figures.

12) Please better clarify if the results in Figure 2 are for stem cells or non-stem cells.

13) In Supplementary Figure 1, the x-axis seems to be mislabeled: instead of % Methylation change (old-young), it should probably be (nonstem-stem).

14) Abstract line 32 should be Jensen

**Authors’ response**

We thank the reviewers for their careful reading of the manuscript and their constructive comments that helped clarify the findings. We have extensively revised the manuscript in response to these comments and provide a point-by-point response below, along with a summary of the changes made to the text.

Reviewer # 1(Reviewer Comment to the Author):

1. With the multiple analysis and sub-analyses, the manuscript becomes difficult to follow for the reader at times. Would benefit from the authors re-assessing readability with a fresh eye. –

We have carefully revised the manuscript and have added more supplementary figures and explanations where necessary.

2. The threshold for RRBS was an appropriate minimum coverage level of 20 reads at each CpG, however, concordance increases with depths >30 [1] - could the authors explore whether higher reads depths show consistency with a representative fraction of their current results? Particularly the effect of only requiring at least 75% of the samples to reach 20 read minimum of the CpGs included in the 4-age group analysis?

**Response: We ran the analysis for differential methylation with coverage >30 and permutation where at least 75% of CpG sites had coverage >30 and results are shown below. As seen from the new figures below there is no change in the outcome of the analysis (no methylation change in stem vs non-stem tissues in young animals, ~5-6% changes in methylated CpG sites with aging in both stem and non-stem tissues, high concordance between aging changes in stem cells and those in non-stem cells; see table below). However, using a coverage > 30 reduces the number of CpG sites analyzed from 125,077 to 70,547 resulting in a 43% reduction in the number of sites analyzed. As a result, we prefer to keep the original analysis at 20 reads but provide *the analysis at 30 reads in the supplementary figure 2, and on lines 146-151.***

Supplementary figure 2: Analysis of stem vs non-stem cell aging at read coverage >30

**Table comparing the change in results between coverage 20 and 30:**

|  | **Coverage 20** | | | **Coverage 30** | | |
| --- | --- | --- | --- | --- | --- | --- |
|  | **Hypermethylation** | **Hypomethylation** | **Hypermethylation** | | **Hypomethylation** |  |
| **Stem vs Non-stem (4 month)** | 0% | 0% | 0% | | 0% |  |
| **Stem vs Non-stem (4 month)** | 0.2% | 0.2% | 0.2% | | 0.2% |  |
| **24-month vs 4-month (stem cells)** | 3% | 1.1% | 4.3% | | 2% |  |
| **24-month vs 4-month (Non-stem cells)** | 2.8% | 0.9% | 3.6% | | 1.4% |  |

|  | Coverage 20 (Spearman coefficient) | Coverage 30 (Spearman coefficient) |
| --- | --- | --- |
| **Stem vs. Non-Stem (USI)** | r = 0.86 | r = 0.88 |
| **Stem vs. Non-Stem (LSI)** | r = 0.84 | r = 0.85 |
| **Stem vs. Non-Stem (COL)** | r = 0.86 | r = 0.91 |

3.It is stated 'significant with age (p-value < 0.05)' on line 90 - is this corrected or nominal?

**Response: The p-value< 0.05 refers to the FDR (< 0.05) corrected p-value that was used for most analysis except for the permutation analysis that uses an empirical p-value. However, upon closer reading of the paper we decided that the said analysis was redundant and have removed it from the manuscript.**

4.Regarding the RNA-seq analysis and the non-promoter CpG island hypermethylation - was there any evidence of changes in expression of non-canonical isoforms with age?

**We looked at non-canonical isoform changes between RNA-seq samples of different ages and did not find any genes that expresses non-canonical isoforms with age. However, we have a limited number of RNA-seq samples per age so we might not have enough power to properly conduct this analysis. *This is now mentioned in the text on line 180-181 and was added to methods section as well.* The figure for the analysis is shown below but due to constrains by genome biology on the number of supplements we will not including it in the paper**

**Figure legend:** MA plots showing the difference in proportions of isoforms per gene vs average expression in 24-month vs 4-month upper small intestine, lower small intestine, and colon samples. Across tissues there were very few genes that showed a significant (FDR < 0.05) difference in isoform usage between 24-month vs 4-month samples.

5. Brief explanation should be included in the Results of what comprises the definition of the 10,366 loci in the Entropy results - so that the reader does not need to go trawling through methods to understand this.

**We apologize for not being clear in the manuscript. The number 10,366 refers to the** **number of unique loci detected across all samples with a coverage greater than 40. For the PCA analysis we used 270 loci detected in all 44 samples which includes stem and nonstem cell samples of all ages (4,12,18 and 24 months). This was due to the nature of PCA analysis requiring all loci be present in all samples. However, for most of the other analysis, we did not require that loci be detected across all samples, thus there was a considerably larger number of loci in the other analysis. *This has now been corrected in the text on line 199-200.***

6.Regarding the DNAm entropy calculated loci - how is this impacted on by CpG density - i.e., is the ageing signal being driven by dense or sparse CpG loci?- look at CpG island vs non island??

**Response: In our data set 69% of the loci analyzed for entropy are located in nonCpG-islands and 31% of the loci are located inside CpG-islands. We divided our analysis into CpG-island and non CpG-island and ran PCA analyses. Results show that both CpG-islands and non CpG-islands clustered with respect to age only. Further analyzing entropy change with age in CpG islands versus non-CpG islands we looked at change in methylation on the y-axis versus change in JSD on the left and CpG islands show more change in methylation and JSD with age when compared to non-CpG island. *This will be added as supplementary figure no 8 and addressed in the paper on lines 221-229.***

**Figure legend:** Change in entropy in CpG islands vs nonCpG islands. (**a-b**) PCA plots constructed using Jensen-Shannon Distances (JSDs) for loci with coverage > 40 in CpG islands (**a**) and nonCpG islands (**b**). Scatterplots showing the change in Jensen-Shannon Distance (JSD) on the x-axis vs change in methylation on y-axis in between stem cells and nonstem cells in small intestine and colon in CpG islands (left) vs non CpG islands(right). (**c-d**) Colon, (**e-f**) Upper small intestine and (**g-h**) Lower small intestine.

7. This is not experimentally required for this paper - but authors should acknowledge in the Discussion that it would clearly be of significant interest if a consistent result was found with the now available Illumina mouse DNAm array - which avoids all the vagaries of stochastic sequencing depth variation.-??

**Response: We agree it would be of significant interest to determine the whether the same dynamics of islands and non-islands and other results we found in our study can be reproduced with illumine mouse arrays or with whole genome sequencing. It would provide more CpG sites for analysis, which could potentially avoid the vagaries of stochastic sequencing and would yield more CpG sites for analysis. However, one drawback in using Illumina mouse arrays is that one cannot measure entropy with array-based methods. *We have added a statement regarding this in our discussion regarding this in line 316-320.***

**Reviewer 2.**

1. Tissue specific differences were analyzed on epithelial cells from colon and small intestine - these closely related cell types/tissues may not be ideal to generalize for tissue-specific differences. Since the authors also analyzed many other tissues, it would be better to consider these for the analysis, too.

**Response: We agree that epithelial cells from the small intestine and colon are very similar and may not be ideal samples to test for tissue specific differences. Following the reviewer’s comments, we analyzed all our tissue samples including (Blood, Heart, Lung, Liver, Spleen, Skeletal Muscle, Kidney, Small intestine, and Colon). We took common CpG sites across all tissues allowing for 25% of the samples to have missing data. We calculated standard deviation for each CpG site across these tissues and took the top 8000 CpG sites with the most deviation and tried to find overlapping CpG sites that are significant with age in our permutation analysis results. Just like in our prior analyses, we found very little overlap between aging-specific and differentiation-specific CpG sites: We found only 104 CpG sites overlapping between the two (~1%) and the result is shown in the Venn diagram below: *It has been added to the analysis in Figure 2h and has been added to the manuscript on lines 139-145***

2) The number of biological replicas should be better indicated in the manuscript. Furthermore, four mouse samples were pooled for RRBS. Is it conceivable that mixing of samples impacts on the JSD if there are interindividual differences?

**Response: The complete list for each organ in the paper and the number of biological replicates is present in the table below. *The list will be submitted as supplementary table 4.***

| Tissue | Age | Number of Biological replicates | Number of animals per pool |
| --- | --- | --- | --- |
| USI (both stem/non-stem) | 4-month | 2 (each pooled) | 4 |
| LSI (both stem/non-stem) | 4-month | 2 (each pooled) | 4 |
| Colon (both stem/non-stem) | 4-month | 2 (each pooled) | 4 |
| USI (both stem/non-stem) | 12-month | 2 (each pooled) | 3 |
| LSI (both stem/non-stem) | 12-month | 2 (each pooled) | 3 |
| Colon (both stem/non-stem) | 12-month | 2 (each pooled) | 3 |
| USI (both stem/non-stem) | 18-month | 2 (each pooled) | 2 |
| LSI (both stem/non-stem) | 18-month | 2 (each pooled) | 2 |
| Colon (both stem/non-stem) | 18-month | 2 (each pooled) | 2 |
| USI (both stem/non-stem) | 24-month | 2 (each pooled) | 2 |
| LSI (both stem/non-stem) | 24-month | 2 (each pooled) | 2 |
| Colon (both stem/non-stem) | 24-month | 2 (each pooled) | 2 |
| Whole Blood | 4-month | 3 | NA |
| Whole Blood | 12-month | 3 | NA |
| Whole Blood | 24-month | 4 | NA |
| Heart | 4-month | 6 | NA |
| Heart | 12-month | 6 | NA |
| Heart | 24-month | 10 | NA |
| Kidney | 4-month | 3 | NA |
| Kidney | 12-month | 3 | NA |
| Kidney | 24-month | 4 | NA |
| Liver | 4-month | 2 | NA |
| Liver | 12-month | 2 | NA |
| Liver | 24-month | 2 | NA |
| Lung | 4-month | 2 | NA |
| Lung | 12-month | 2 | NA |
| Lung | 24-month | 2 | NA |
| Skeletal Muscle | 4-month | 2 | NA |
| Skeletal Muscle | 12-month | 2 | NA |
| Skeletal Muscle | 24-month | 2 | NA |
| Spleen | 4-month | 2 | NA |
| Spleen | 12-month | 2 | NA |
| Spleen | 24-month | 2 | NA |

**To look at whether pooling affects the data. We looked at data where we have samples from individual mice and bioinformatically pooled samples to mimic pooling. We took samples from lungs, which is our most proliferative tissue besides the intestine, and heart which is the least proliferative tissue. Results show that pooling more samples decreases JSD instead of increasing it, and increasing the number of samples pooled resulted in a decrease in JSD. As a result, our data on pooled samples is possibly an underestimate of the true differences between individuals. *We have included this conclusion in line 259-264.* Due to the constraints of number of supplementary figures we can submit to genome biology we will not be including this in supplementary data.**

FIGURE LEGEND: Comparison of JSD in pooled versus non-pooled sample. **a-b**. Violin plots of JSD values (x-axis) and average % methylation (y-axis) in pooled and not pooled samples in the lung (**a**) and heart (**b**)

3) The concept of JSD needs to be much better clarified in the manuscript - particularly since this is the most innovative aspect. Looking at other studies I got the impression that JSD is typically higher at *hemimethylated sites (peaking at 50% methylation level) whereas it is generally lower at none or highly* methylated sites. If so, this needs to be better clarified and taken into consideration. It is known that age-related CpGs are often hemimethylated, while tissue-specific DNA methylation may be more at the extremes. This might at least contribute to the observed discrepancy in JSD.

**Response: We acknowledge that Jensen Shannon Distance (JSD) is not the current standard method to quantify entropy in the field, and it differs from Shannon’s entropy, which could be creating some confusion. We would like to clarify that Jensen Shannon Distance (JSD) is a method of calculating entropy with a distance metric using a reference** **sample**. **If the reference sample and test sample distributions don’t overlap at all then JSD equals 1(which is the maximum), if they overlap completely then JSD equals 0, and with intermediate levels of overlap the value ranges between 0 and 1. *This explanation has been included in the manuscript in lines 188-196.***

**Example 1: Case of low JSD change between test sample and reference distribution**

**Example 2: Case of high JSD change between test sample and reference distribution**

**Thus, a change in the methylation distribution from the reference sample results in increasing JSD.**

**More conventional methods of quantifying entropy such as Shannon’s entropy do not consider distribution and direction of methylation change, resulting in the possibility of the alleles having the same number of fully methylated or unmethylated CpG’s but a very different distribution. For example (figure below), when alleles from the young sample are unmethylated whereas alleles from the old sample are fully methylated, the value of Shannon’s entropy between the samples will be the same but JSD shows a high difference. Therefore, we think JSD gives a better measure of age-related entropy.**

**As an example, we calculated Shannon’s entropy for a locus with high change in JSD (example below) between reference and test sample distribution and the change in Shannon’s entropy is very small, only 0.08, whereas the change in JSD is quite large at 0.65.**

**More details on this are in the reference paper :**

Jenkinson G, Abante J, Feinberg AP, Goutsias J. An information-theoretic approach to the modeling and analysis of whole-genome bisulfite sequencing data. BMC Bioinformatics. 2018;19(1)

4) The reference distribution for calculation of JSD should be better clarified. Maybe in the manuscript??

**Response: Reference distribution for calculation of JSD is the average JSD of young samples from the corresponding tissue. *This is mentioned in line 193-194.***

5) It is unclear how tissue renewal rates were measured (e.g., in Figure 5c). Blood is a tissue with enormous turnover. I am surprised to find the tissue renewal rate/30% for blood at the lower edge of all tissues.

**Response: We calculated the turnover rate for blood using the turnover rate for hematopoietic stem cells. Then using that we calculated turnover rate per 30 days using the formula (30-day turnover rate = 30 X average turnover rate per day). Our data for small intestine and colon shows that methylation change with age is a stem cell turnover phenomenon. Thus, when data was available for stem cell turnover, we used those numbers. If not available we used the turnover rates observed for whole organs. The table with the observed turnover time and the calculated number of turnover times per 30 days is listed in the table below. *We have provided the table in Supplementary table 3 added updated it in the manuscript in lines 250-257.***

| **Organ/Tissue** | **Turnover time** | **No. of turnover**  **/30 days** | **Method** | **Reference** |
| --- | --- | --- | --- | --- |
| Small intestine | 2-3 days | 10 | 3H-thymidine | Potten [33] |
| Colon | 3-4 days | 8.5 | 3H-thymidine | Potten [33] |
| Blood (Hematopoietic Stem cells) | 57 days | 0.52 | Bromodeoxyuridine | Chesier [34] |
| Liver Hepatocytes | 200-400 days | 0.1 | 3H-thymidine | Magami et. al [35] |
| Lung | Bronchial epithelium  (2-7 days)  Bronchiolar epithelium-(10 days)  Alveolar epithelium-  (28-35 days)  Average =10.5 days | 3.53 | 3H-thymidine | Bowden [36] |
| Striated muscle (satellite cells)  (Rat muscle) | >170 days | 0.17 | 3H-thymidine | Allbrook et. al [37] |
| Spleen (whole) | 9 days | 3.3 | Bromodeoxyuridine | Crippen et. al [38] |
| Kidney (renal corpuscle and the nephron tubule) | >120 days | 0.001 | 3H-thymidine | Litvak & Baserga [39] |
| Cardiomyocytes | >300 days | 0.001 | 3H-thymidine | Cluzeaut & Maurer-Schultze [40] |

6) On the other hand, there is a bigger hierarchy in the hematopoietic system between the quiescent long-term repopulating hematopoietic stem cells and the various intermediate faster proliferating progenitor cells. Separation in stem cells and none-stem cells might be too simplistic for many tissues (that even comprise multiple cell types).

**Response: We acknowledge that there is a limitation in our analysis because in the colon and small intestine the hierarchy is simple whereas other tissues such as blood have a more complex hierarchy. It is possible that in tissues with complex hierarchy there might be slightly different results and it is worth examining in the future. *We have added this to the discussion in line 308-310.***

7) Colon stem cells in Figure 5 seem to be on the same line as non-stem cells. This might argue against a clear finding toward their hypothesis. Why are renewal rates anticipated similar in stem cells and non-stem cells? Why does Figure 5a only depict COL and not SI?-> supplement

**Response: Indeed, we find that Colon stem cells are on the same line as non-stem cells. Our hypothesis is that methylation drift with age is a stem cell turnover phenomenon and that the DNA methylation changes in stem cells are inherited by the non-stem cells; therefore we expected that nonstem cells would have almost identical JSD as the stem cells they are derived from. As a result, the data result we found is consistent with our hypothesis. In the process of differentiation there is no resetting of the epigenome and they do not divide much further, as a result their renewal rate history is the same as that of the stem cell. Consequently, we used the renewal rate of the stem cell for this analysis. *We have now included the figure for small intestine (below) as a part of supplementary figure 10 and included it in the manuscript in line 244-245.***

8) It is well known that even non-proliferative tissues, such as brain, have extensive age-associated DNA methylation. As indicated, this has been shown by many functional signatures. Furthermore, multi-tissue clocks have been successfully established. It therefore appears surprising that their data suggest very little overlap in aging changes between distantly related tissues and between tissues that have very different stem cell proliferation rates.

**Response: The existence of methylation clocks in non-proliferative tissues is an important topic and we acknowledge that there are reported multi tissue clocks in the literature. A full discussion of the advantages and drawbacks of these clocks is beyond the scope of this paper. However, because this is an important issue, we wanted to use our data to ask that question. In our data the most non-proliferative tissue is Heart and we find very little methylation changes there (Heart- figure below). In general, the most non-proliferative tissues that can be easily examined are heart and skeletal muscle (glial cell contamination makes analyzing brain more difficult in this regard). To confirm our own data, we reanalyzed published data on another non-proliferative tissue (Skeletal muscle, Thompson *et. al*) and do not find much DNA methylation change with age. The small changes observed (see below) could easily be due to a small amount of contamination of the tissues with proliferative cells (e.g. blood).**

**a**. Volcano plots showing methylation differences between young (4 month) and old (24 month) samples in heart. **b**. Volcano plots showing methylation differences between young (2 month) and old (20 month) samples in skeletal muscle methylation data obtained from Thompson et. al.

Thompson MJ, Chwiałkowska K, Rubbi L, Lusis AJ, Davis RC, Srivastava A, Korstanje R, Churchill GA, Horvath S, Pellegrini M. A multi-tissue full lifespan epigenetic clock for mice. Aging (Albany NY). 2018 Oct 21;10(10):2832-2854. doi: 10.18632/aging.101590. PMID: 30348905; PMCID: PMC6224226.

**The difference between our data and the published literature might be due to contamination of tissues with proliferative cells. For example, the brain consists of glial cells which are proliferative, thus it is expected to find age related changes in the brain. In addition, the brain has a high level of DNA methyl transferases (DNMT’s) in quiescent tissues (neurons), thus it is possible that neurons are undergoing DNA methylation change due to other reasons. It is also possible that some of the changes reported in the literature in non-proliferative tissues may be detecting changes in cell composition with age rather than changes in DNA methylation with age. Since we currently do not have data on this, we chose to focus the results and discussion on the data that we do have.**

Tognini P, Napoli D, Pizzorusso T. Dynamic DNA methylation in the brain: a new epigenetic mark for experience-dependent plasticity. Front Cell Neurosci. 2015 Aug 25;9:331. doi: 10.3389/fncel.2015.00331. PMID: 26379502; PMCID: PMC4548453.

9. The threefold change was observed in both stem and non-stem cells of 24 months (Figure 5a). How do they conclude that this is due to stem cell division (line 212 as well as line 249-250)? As it stands, there appear to be hardly conclusive results how stem cell division or cell division rate are related to the epigenetic drift

**Response: We came to this conclusion because there was very little difference between stem cells and non-stem cells from the same age (figure below). The three-fold difference seen in fig 5a is between 4-month and 24-month sample. As discussed earlier, differentiated cells inherit DNA methylation patterns from stem cells and thus have a nearly identical proliferation history. *We have now included the stem vs. nonstem comparison for upper small intestine, lower small intestine and colon as a part of supplementary figure 6 and included it in the manuscript in line 206-208.***

Figure legend: Entropy change between stem and nonstem cells in the small intestine and colon***.*** Scatterplots showing the change in Jensen-Shannon Distance (JSD) on the x-axis vs change in methylation on y-axis, between (left) young and old stem cells (Lgr5-GFP+), and (right) young and old nonstem cells (Lgr5-GFP-) in (**g**) Upper small intestine (USI), (**h**) Lower small intestine (LSI) and (**i**) colon (COL).

Minor:
10) The GEO ID has not yet been provided.

**Response**: Data has been uploaded on GEO (accession number: GSE213723. <https://www.ncbi.nlm.nih.gov/geo/query/acc.cgi?acc=GSE213723>) and a data availability section has ben added to the paper

**2^nd^ round**

**Reviewer 1**

>>>Reviewer 1 Replies

1. With the multiple analysis and sub-analyses, the manuscript becomes difficult to follow for the reader at times. Would benefit from the authors re-assessing readability with a fresh eye.

Response: We have carefully revised the manuscript and have added more supplementary figures and explanations where necessary.

>>> Great

2. The threshold for RRBS was an appropriate minimum coverage level of 20 reads at each CpG, however, concordance increases with depths >30 [1] - could the authors explore whether higher reads depths show consistency with a representative fraction of their current results? Particularly the effect of only requiring at least 75% of the samples to reach 20 read minimum of the CpGs included in the 4-age group analysis?

Response: We ran the analysis for differential methylation with coverage >30 and permutation where at least 75% of CpG sites had coverage >30 and results are shown below. As seen from the new figures below there is no change in the outcome of the analysis (no methylation change in stem vs non-stem tissues in young animals, ~5-6% changes in methylated CpG sites with aging in both stem and non-stem tissues, high concordance between aging changes in stem cells and those in non-stem cells; see table below). However, using a coverage > 30 reduces the number of CpG sites analyzed from 125,077 to 70,547 resulting in a 43% reduction in the number of sites analyzed. As a result, we prefer to keep the original analysis at 20 reads but provide the analysis at 30 reads in the supplementary figure 2, and on lines 146-151.

>>> OK great

3. It is stated 'significant with age (p-value < 0.05)' on line 90 - is this corrected or nominal?

Response: The p-value< 0.05 refers to the FDR (< 0.05) corrected p-value that was used for most analysis except for the permutation analysis that uses an empirical p-value. However, upon closer reading of the paper we decided that the said analysis was redundant and have removed it from the manuscript.

>>> Ok

4. Regarding the RNA-seq analysis and the non-promoter CpG island hypermethylation - was there any evidence of changes in expression of non-canonical isoforms with age?

Response: We looked at non-canonical isoform changes between RNA-seq samples of different ages and did not find any genes that expresses non-canonical isoforms with age. However, we have a limited number of RNA-seq samples per age so we might not have enough power to properly conduct this analysis. This is now mentioned in the text on line 180-181 and was added to methods section as well. The figure for the analysis is shown below but due to constrains by genome biology on the number of supplements we will not including it in the paper

>>> OK

5. Brief explanation should be included in the Results of what comprises the definition of the 10,366 loci in the Entropy results - so that the reader does not need to go trawling through methods to understand this.

Response: We apologize for not being clear in the manuscript. The number 10,366 refers to the number of unique loci detected across all samples with a coverage greater than 40. For the PCA analysis we used 270 loci detected in all 44 samples which includes stem and nonstem cell samples of all ages (4,12,18 and 24 months). This was due to the nature of PCA analysis requiring all loci be present in all samples. However, for most of the other analysis, we did not require that loci be detected across all samples, thus there was a considerably larger number of loci in the other analysis. This has now been corrected in the text on line 199-200.

>>> OK

6.Regarding the DNAm entropy calculated loci - how is this impacted on by CpG density - i.e., is the ageing signal being driven by dense or sparse CpG loci?- look at CpG island vs non island

Response: In our data set 69% of the loci analyzed for entropy are located in nonCpG-islands and 31% of the loci are located inside CpG-islands. We divided our analysis into CpG-island and non CpG-island and ran PCA analyses. Results show that both CpG-islands and non CpG-islands clustered with respect to age only. Further analyzing entropy change with age in CpG islands versus non-CpG islands we looked at change in methylation on the y-axis versus change in JSD on the left and CpG islands show more change in methylation and JSD with age when compared to non-CpG island. This will be added as supplementary figure no 8 and addressed in the paper on lines 221-229.

>>> OK

7. This is not experimentally required for this paper - but authors should acknowledge in the Discussion that it would clearly be of significant interest if a consistent result was found with the now available Illumina mouse DNAm array - which avoids all the vagaries of stochastic sequencing depth variation.

Response: We agree it would be of significant interest to determine the whether the same dynamics of islands and non-islands and other results we found in our study can be reproduced with illumine mouse arrays or with whole genome sequencing. It would provide more CpG sites for analysis, which could potentially avoid the vagaries of stochastic sequencing and would yield more CpG sites for analysis. However, one drawback in using Illumina mouse arrays is that one cannot measure entropy with array-based methods. We have added a statement regarding this in our discussion regarding this in line 316-320.

>>> OK

**Reviewer 2**

The authors have revised their manuscript and addressed each of my comments. Overall, I believe that the concept is innovative, but it is not an easy read and I still have the impression that some findings are overstated.
- Their new method of entropy measurement by Jensen Shannon Distance (JSD) is different to Shannon´s Entropy, which is more commonly used. While this is now mentioned in few lines in the results section, the reader might be confused. The authors gave a better explanation in their rebuttal letter than in the text. A comparison of the results with Shannon´s Entropy might also be useful.
- Their statement that “while DNA methylation overall is primarily tissue-specific, DNA methylation entropy is exclusively an aging phenomenon.” (line 204) seems to be primarily derived from the PCA results in figure 4C.
- I have the impression that the statement that “DNA methylation drift and increased entropy with age is primarily caused by, and is a sensor for, stem cell replication in adult tissues.” (abstract) is mainly due the data points of colon and small intestine.
- Figure 1 appears to be completely wrong: Figure 1a is no scheme, the difference of figure 1a and 1b is not clear. Figure 1c-e is identical with figure 3a-c.
- In figure 5 the panels b and c are confused.
- There are many small typos and inconsistencies throughout the manuscript: Line 69: "separate tissues"; In line 193 they now indicate the samples for their reference distribution “(average JSD in young samples in our)” – probably they refer to the 4-months mice of corresponding tissue, but this important point might be stated more clearly; Line 262 “show”; Line 297 “of methylation analysis”; line 318: “to see if”.

**Authors’ response**

We thank the reviewer for the comments and suggestions. Here are our responses:

*Their new method of entropy measurement by Jensen Shannon Distance (JSD) is different to Shannon´s Entropy, which is more commonly used. While this is now mentioned in few lines in the results section, the reader might be confused. The authors gave a better explanation in their rebuttal letter than in the text. A comparison of the results with Shannon´s Entropy might also be useful.*

**Response:** We have included a supplementary methods section that mirrors the previous rebuttal letter. In addition, supplementary figure 10 was updated with figures comparing changes in Shannon’s entropy between 4 month and 24-month sample in colon stem cells, colon nonstem cells, heart, liver, kidney, and spleen and this is now included in the paper in line 192-194. Essentially, JSD shows significant changes while Shannon’s entropy does not.

-	Their statement that “while DNA methylation overall is primarily tissue-specific, DNA methylation entropy is exclusively an aging phenomenon.” (line 204) seems to be primarily derived from the PCA results in figure 4C.

**Response:** We came to that conclusion by comparing the associations between age and tissue for both standard DNA methylation (Figure 1b) and DNA methylation entropy (Figure 4C). If you consider the PCA in Figure 1b constructed using standard DNA methylation, the primary variation in the data (PC1) is explained by tissue type as can be seen in the clusters there. In contrast the PCA in Figure 4C, constructed using methylation entropy, shows that age explains the primary variation in the data (PC1). **This was addressed in the discussion section 302-307.**

-	I have the impression that the statement that “DNA methylation drift and increased entropy with age is primarily caused by, and is a sensor for, stem cell replication in adult tissues.” (abstract) is mainly due the data points of colon and small intestine.

**Response:** We respectfully disagree. We primarily came to this conclusion with data from three main types of tissues – non-proliferative (best example being heart), mildly proliferative (blood and lung for example) and very highly proliferative (colon and small intestinal epithelium). Aligning these is sufficient to give the strong correlation we observe, and our conclusion. Not all tissues fit neatly in Figure 5b obviously, but this likely reflects biology and unknowns. For example, liver can be quiescent or proliferative depending on exposures over a lifetime. In some cases, the methylation data can be more accurate than the reported tissue renewal rates (blood and spleen likely have a shared ultimate stem cell, explaining similar methylation but different reported turnover rates). Also, methylation data for the organs was generated using whole organs and not purified cell types like epithelium. Some organs are constituted of cell types derived from different stem cells leading to skewing of results. **This has been addressed in the discussion section line 311-323.**

-	Figure 1 appears to be completely wrong: Figure 1a is no scheme, the difference of figure 1a and 1b is not clear.

Figure 1c-e is identical with figure 3a-c.

**Response:** We apologize for this confusion. The wrong file was uploaded. This has been corrected

-	In figure 5 the panels b and c are confused.

**Response:** We have fixed this error.

-	There are many small typos and inconsistencies throughout the manuscript: Line 69: "separate tissues"; In line 193 they now indicate the samples for their reference distribution “(average JSD in young samples in our)” – probably they refer to the 4-months mice of corresponding tissue, but this important point might be stated more clearly; Line 262 “show”; Line 297 “of methylation analysis”; line 318: “to see if”.

**Response:** We have corrected the above-mentioned errors.
